# Correction: The moral significance of protecting environmental and cultural objects

**DOI:** 10.1371/journal.pone.0300942

**Published:** 2024-03-22

**Authors:** The PLOS ONE Staff

## Notice of republication

This article was republished on March 5, 2024, to rectify a spelling error in the title that occurred during the typesetting process. The publisher apologizes for this error. Please download this article again to view the correct version. The originally published, uncorrected article and the republished, corrected articles are provided here for reference.

## Supporting information

S1 File Originally published, uncorrected article. (PDF)

S2 File Republished, corrected article. (PDF)
