# Supplementary material for: The moral significance of protecting environmental and cultural objects
Source: PLoS One. 2023 Feb 9;18(2):e0280393. doi: 10.1371/journal.pone.0280393 (PMC9910731; doi:10.1371/journal.pone.0280393)
Supplement: S1 File — (DOCX) [file pone.0280393.s001.docx]

**SI File – Supporting information**

# Pilot 1

In order to develop a focal list of entities for our studies, we first sought to determine the kinds of objects people value in two different ways – because they possess intrinsic value vs. extrinsic value. Fifty-three participants from Amazon’s Mechanical Turk completed the survey online in exchange for a small payment (27 female, *Mage* = 33.13, *SD =* 12.53, all native English speakers). Participants were informed that they would be contemplating the different ways that we can value things in the world around us, and that they would be asked to produce a list of things that we can value in two different ways. We next provided them with a definition of intrinsic and extrinsic value (we note that in later studies we used a sparser definition of these two forms of value).

For intrinsic value, participants read:

*There are many things in the world that we value in different ways. For instance, some things we value in their own right. They are not valuable because they can do something for us or provide something for us, they are simply valuable for their own sake. This kind of value is often referred to as intrinsic value. A good indicator of whether or not something has intrinsic value for us is that it could not be easily replaced if it were destroyed. This is because the thing itself has a specific meaning to us.*

For extrinsic value, participants read:

*Another way that we can value something is because it provides an effective means to an end – it is valuable for achieving something else that we value, for instance a goal that we have. This kind of value is referred to as extrinsic value (or perhaps instrumental value). A good indicator of whether or not something has extrinsic value for us is whether we believe it could be easily replaced if it were destroyed. This is because the thing itself has no specific meaning to us.*

Immediately following these definitions, participants were required to generate a list of things that were primarily valuable to them because of their intrinsic or extrinsic value. During this part of the survey we prevented participants from moving onto the next page for one minute to ensure that they would take their time and carefully consider their responses. We also encouraged them to take longer than the one minute minimum and to generate as many items as possible. Finally, we asked participants to indicate how clearly they understood the terms “intrinsic value” and “extrinsic value” (0 = *not at all*, to 10 = *very much so*).

## Results and Discussion

One-sample *t*-tests indicated that participants clearly understood the meaning of intrinsic (*M* = 9.96, *SD* = 1.63) and extrinsic value (*M* = 9.96, *SD* = 1.60) scoring well above the midpoint of the scale (both *p* values < .001). The most common responses were (*intrinsic value*: family/friends, photos/pictures, rings, health, memories; *extrinsic value*: car, phone, computer, television, clothes), and the most common categories were (*intrinsic value*: abstract values, mementos, photos, jewelry, family; *extrinsic value*: cars, household items, electronic items, clothing, money). From these responses we generated a list of objects that were commonly considered to possess either intrinsic or extrinsic value. We focused specifically on objects as this was the focus of our analysis, and so did not incorporate concepts related to abstract values or items such as memories. To this list we also added some additional targets that we felt were particularly distinctive exemplars of intrinsic vs. extrinsic value, or that provided explicit examples of frequently mentioned categories. In developing our list, we attempted to match objects across both types of value. For instance, an original artwork (intrinsic value), a copy of an artwork (extrinsic value), a sea turtle (intrinsic value), sea turtle meat (extrinsic value). This resulted in a total of twenty objects (ten with intrinsic value, ten with extrinsic value; see Table A).

# Pilot 2

Having compiled a list of objects ostensibly possessing either intrinsic or extrinsic value in Pilot Study 1, we sought to validate this list in Pilot 2 by asking participants to rate each object according to our existing definitions of intrinsic and extrinsic value. Fifty-seven participants (41 male, *Mage* = 27.70, *SD =* 6.91, all native English speakers) from Amazon’s Mechanical Turk completed the survey online in exchange for a small payment. Participants were presented with the definitions of intrinsic vs. extrinsic value, followed by the list of twenty objects developed from Pilot 1, rating the extent to which each object possessed intrinsic vs. extrinsic value on an eleven-point scale (-5 = *more intrinsic value*, to 0 = *neutral,* to +5 = *more extrinsic value*). After making their ratings, participants indicated how clearly they understood what the terms “intrinsic value” and “extrinsic value” meant, using an eleven point Likert-type scale (0 = *not at all*, to 10 = *very much so*).

## Results and Discussion

Consistent with Pilot 1, one sample *t*-tests indicated that participants clearly understood the meaning of both intrinsic (*M* = 9.98, *SD* = 1.34) and extrinsic value (*M* = 9.79, *SD* = 1.86) scoring well above the midpoint of each scale (both *p* values < .001).

Single sample *t*-tests were then performed to examine whether the objects that we generated from the Pilot were rated as intrinsically or extrinsically valuable (values significantly less than zero indicated intrinsic value; values significantly greater than zero indicated extrinsic value). On average, participants rated the intrinsic exemplars (*M* = -2.61, *SD* = 1.73) as intrinsically valuable, *t*(56) = -11.38, *p* < .001 (one-tailed against the scale midpoint). Ratings across the list of intrinsic exemplars had high internal consistency (Cronbach’s alpha = .83) although reliability was lower for extrinsic objects (α = .49). Individual t-tests revealed that each intrinsically valuable exemplar received a rating significantly less than the zero mid-point (means ranged from -1.18 to -3.77, all *p* values < .001, except for diverse ecosystem, *p* = .002, and sea turtle, *p* = .004). On average, participants rated the extrinsic exemplars (*M* = 1.35, *SD* = 1.31) as extrinsically valuable, *t*(56) = 7.76, *p* < .001 (one-tailed against the scale midpoint). Individual t-tests revealed that most of the extrinsic exemplars received a rating significantly greater than the zero mid-point (means ranged from -0.79 to 3.09, all *p* values < .05, with the exception of tree plantation, *p* = .18, and a copy of something original, *p* = .59). These ratings validated the list of entities developed in the pilot study as exemplars that separately tap intrinsic and extrinsic value.

## Additional Ratings and Results

As part of Pilot 2 we developed a list of fourteen attributes that we believed captured the qualities which afford certain entities high vs. low levels of intrinsic value. There were seven attributes thought to be associated with high intrinsic value (old, personal, unique, precious, hard to replace, hard to reproduce, hard to repair), and seven attributes thought to be associated with low intrinsic value (new, public, common, useful, easy to replace, easy to reproduce, easy to repair). Fifty-seven participants (41 male, *Mage* = 27.70, *SD =* 6.91, all native English speakers) from Amazon’s Mechanical Turk rated these attributes on the extent to which each would likely be used to describe an object that holds intrinsic vs. extrinsic value (-5 = *intrinsic value*, 0 = *neutral*, 5 = *extrinsic value*). This revealed that the high intrinsic attributes were rated as associated with intrinsic value (*M =* -3.08, *SD* = 1.80, α = .86, *t*(50) = -12.22, *p* < .001, one-tailed against the 0 midpoint), and the low intrinsic attributes were associated with extrinsic value (*M* = 2.89, *SD* = 1.43, α = .73, *t*(50) = 14.46, *p* < .001, one-tailed against the 0 midpoint).

# Pilot 3

For Pilot 3 our aim was to develop six subcategories of intrinsic value (and corresponding categories of extrinsic value), and to have participants generate exemplars (e.g., entities/objects) that were representative of each of these subcategories. To achieve this, we drew on the attributes from Pilot 2 that had the highest ratings of intrinsic value (*personal, old* and *unique* – changed to *rare*). We added to this list two attributes that featured in the literature on moral value (*mind* and *sacredness*). We also added to this list one additional attribute (*beautiful*). We then developed a corresponding list of extrinsically valuable sub-categories. This resulted in 6 dimensions (*beautiful* vs. *ugly*, *rare* vs. *common*, *old* vs. *new*, *personal* vs. *public*, *sacred* vs. *secular*, and *mindful* vs. *non-mindful*).

Sixty-three participants (32 female, *Mage* = 36.35, *SD =* 12.93, 61 native English speakers) from Amazon’s Mechanical Turk took part in the study. To begin, participants were presented with the following instructions:

*“We often distinguish things/objects/entities in the world around us because they possess a variety of different and distinct qualities (e.g., beautiful or old). In this study we would like you to generate a short list of things/objects/entities that are known for possessing a number of these select qualities.”*

The six dimensions were presented to participants in random order. For each dimension, participants were required to think of five things/objects/entities in the world around us that are characteristic of the various intrinsic qualities (e.g., things that are beautiful), as well as five things that are characteristically counter to these qualities (e.g., things that are ugly). For each of the twelve sub-categories, the ten most popular responses were gathered. From these lists, the researchers then selected what they deemed to be the five most suitable and distinct exemplars across the categories and resolved any disagreements through discussion. This produced a total of sixty exemplars that were selected for analysis in the main study (see Table B).

# Study 1

## Inter-correlations between all variables

The inter-correlations among perceived intrinsic value, extrinsic values, moral capacity/sentience, the extent to which the destruction of the entity might negatively impact the welfare of others, and the wrongness to destroy the entity, for both intrinsically valuable and the extrinsically valuable entities are presented in Table B.

## Additional Results

For completeness, we also examined what predicts the wrongness of destroying extrinsically valuable entities using averaged z-score variables. The overall model was significant, *F* (6, 91) = 24.42, *p* < .001, *R^2^* = .63. Judgements of whether it would matter to other people if the entities were destroyed, β = .45, *p* < .001, and that the entities possessed intrinsic value, β = .29, *p* = .001 were again significant predictors, as with the intrinsically valuable exemplars. This time the extent to which an entity could experience pain was also a significant predictor, β = .34, *p* = .017. Ratings of whether destruction would matter to the entity itself, β = -.08, *p* = .567, or would negatively affect its interests, β = .07, *p* = .358, and ratings of extrinsic value, β = .06, *p* = .435 were non-significant predictors.

# Study 2

The perceived wrongness to destroy the intrinsic exemplars was positively correlated with the perceived wrongness to destroy the extrinsic exemplars, *r*(101) = .68, *p* < .001.

# Study 3

## Inter-correlations between all variables

The inter-correlations among perceived intrinsic value, extrinsic value, economic value, usefulness, and the wrongness to destroy the entity, for both intrinsically valuable and the extrinsically valuable entities are presented in Table C.

## Additional Results

For completeness, we also examined what predicts the wrongness of destroying extrinsically valuable entities. The model was significant, *F* (4, 104) = 10.53, *p* < .001, *R^2^* = .29. As with the intrinsic exemplars, judgements of whether the entities possessed intrinsic value, β = .31, *p* < .001, was a significant predictor in the model. This time, however, so were judgements of economic value, β = .21, *p* = .028, and usefulness, β = .32, *p* = .001. Extrinsic value was a significant, although negative predictor, β = -.20, *p* = .038.

# Study 4

## Additional Results

Prior to comparing “Intrinsic value” against specific source of value we also ran simultaneous multiple regressions including all sources of value. When all predictors were entered into a regression predicting “How wrong would it be to destroy…”, intrinsic value made a significant independent contribution (β = .52, *p* = .019). Intrinsic value did not make a significant independent contribution when predicting “How much money would you require to destroy…” (β = .26, *p* = .392). However, given high correlations between all predictors, multicollinearity was a problem with VIF values ranging between 2.3 and 27.5. For this reason, we decided to focus on separate regressions contrasting “Intrinsic value” with each of the other five qualities separately. This allowed us to assess whether, when compared to each alternative quality, intrinsic value remained an independent predictor and whether it also explained more variance – for both the wrongness of destroying variable and the money required to destroy variable.

# Study 5

Participants were presented with a picture of one of four entities: an old growth forest, a cricket, a gemstone, or a table. Under each image was a short believable but partially fictional description, which described the entity as either intrinsically or extrinsically valuable. We asked participants to read the descriptions carefully and told them that they would be answering some questions about the object/entity later. To prevent participants from skipping through the descriptions, the descriptions remained on the screen for one minute before participants could move onto the next page. The definitions were as follows:

Old Growth Forest (see Figure A): Intrinsic value condition

*Old growth forests are natural forests that have developed undisturbed over vast stretches of time. These unique forests are home to some of the oldest trees on earth, with many in excess of 120 years and some as old as 1000. Old growth trees betray their advanced age with antique bark that is deeply furrowed or shaggy. These beautiful forests are notoriously slow developers, taking up to 400 years to regenerate. They are also rare: of all the forests on earth, just 1% meet the definition of old growth.*

*
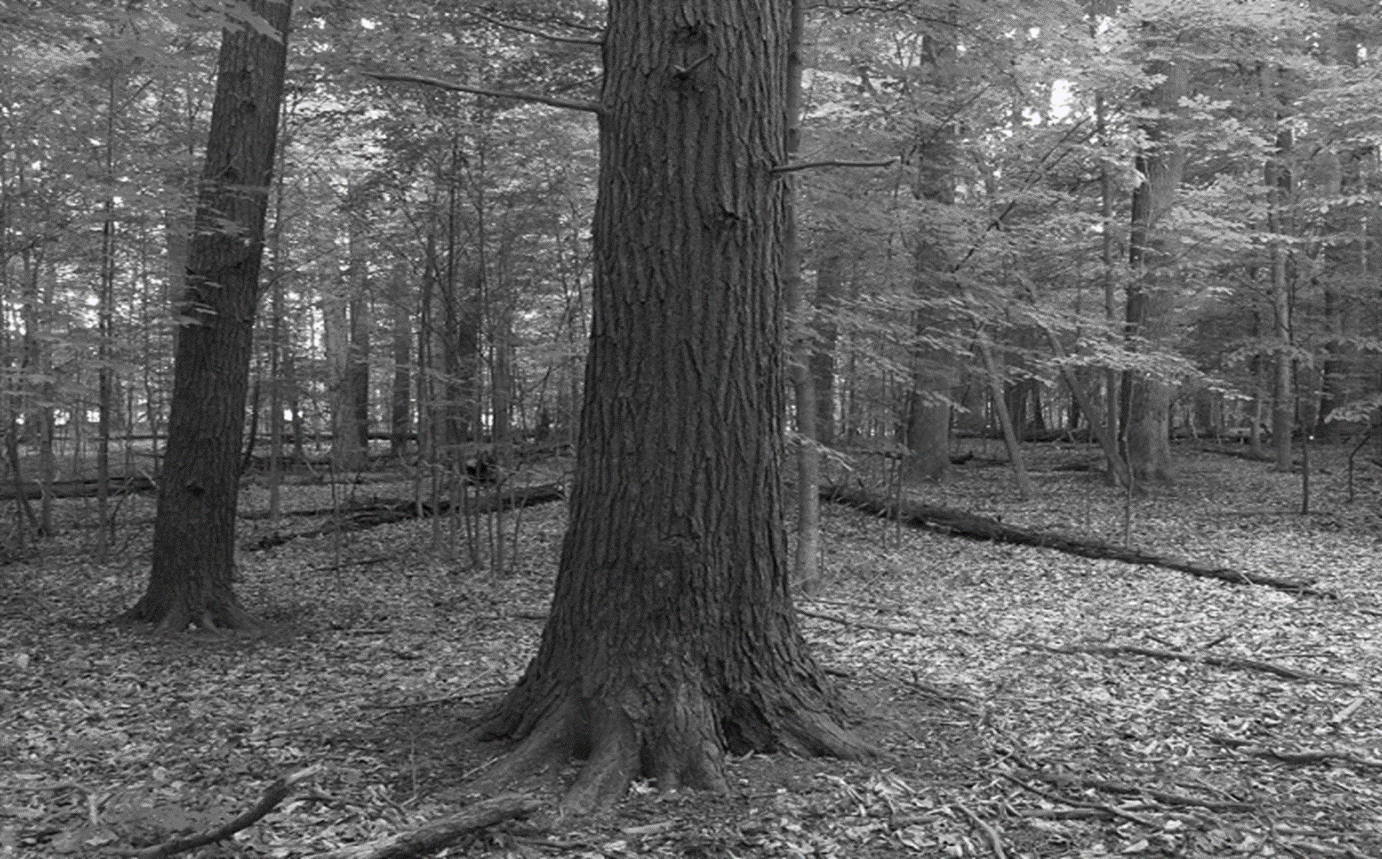
*

**Figure A. Picture of old growth forest used in the study.**

Extrinsic value condition

*Old growth forests are natural forests that have developed undisturbed over vast stretches of time. These forests serve a number of important functions: they soak up carbon dioxide from the atmosphere, they help regulate the climate, and they provide a home for a vast number of flora and fauna, thus helping to maintain biodiversity. Old growth forests also have great monetary value; each year, they generate over one billion dollars from tourism alone. Owing to their high quality timber, old growth forests also contribute substantially to the economy through the logging industry.*

Cricket (see Figure B): Intrinsic value condition

*Crickets are small insects with short, cylindrical bodies, round heads, and long antennae. Despite their tiny brains, crickets display complex and intelligent behaviour. They navigate over long distances, search for food, avoid predators, communicate with each other, display courtship, and care for their young. The complexity of their behavioural repertoire is comparable to any mammal, and some scientists have suggested that they may even have consciousness.*

*
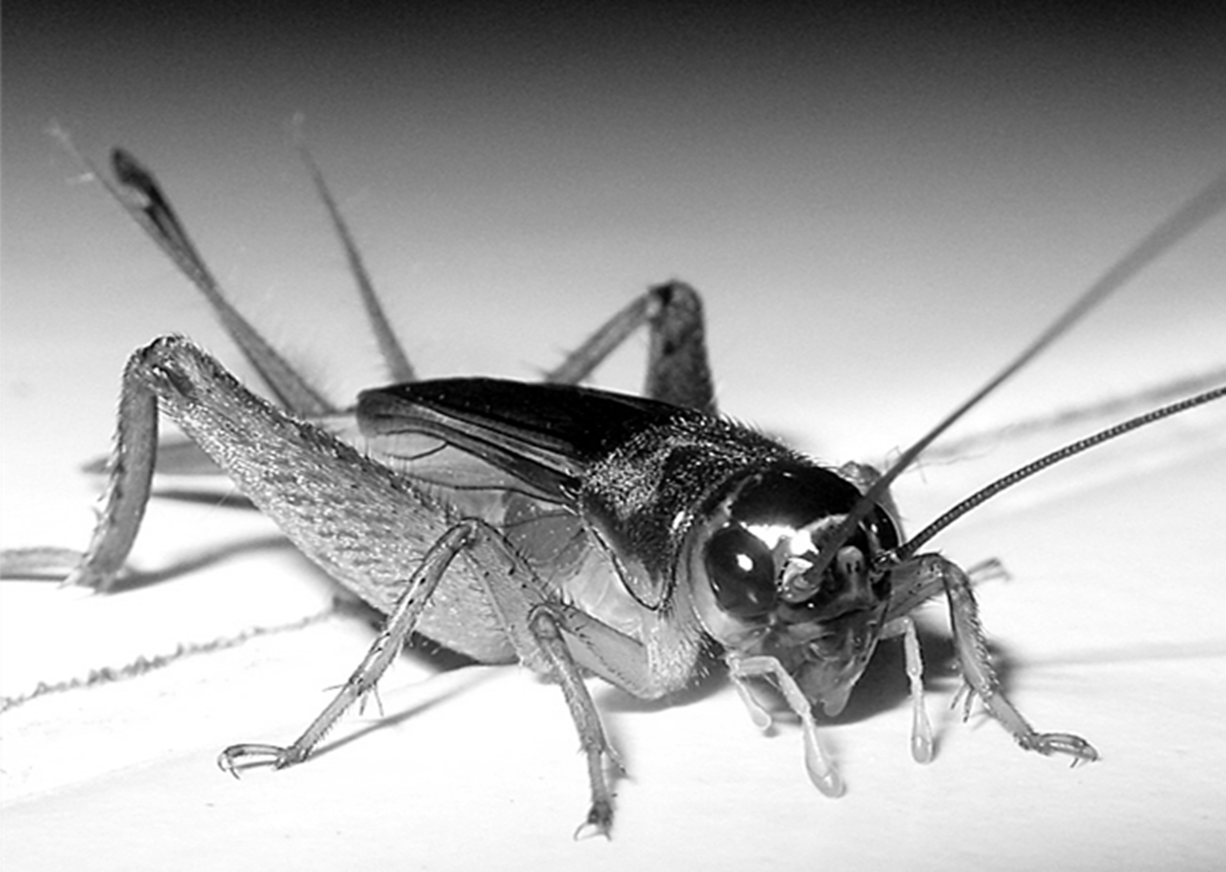
*

**Figure B. Picture of cricket used in the study.**

Extrinsic value condition

*Crickets are small insects with short, cylindrical bodies, round heads, and long antennae. Crickets are surprisingly useful. They are used extensively as food for zoo and laboratory animals, and they are even reared for human consumption in a number of countries. Crickets also play a vital role in many ecosystems. They consume large quantities of highly resistant, cellulose-rich plant materials, and produce fecal pellets that are easily decomposed by bacteria and fungi.*

Gemstone (see Figure C): Intrinsic value condition

*Found only in the foothills of Mount Kilimanjaro in Northern Tanzania, arilaete is one of the rarest and most beautiful gemstones on earth. The stone is renowned for its striking blue/purple appearance and its strange optic properties – it can actually change colour depending on what kind of light it’s under. This property is due to an exceedingly rare combination of minerals that includes titanium, iron, and chromium. Arilaete’s scarcity greatly exceeds that of any diamond, and as of 2014 there were just 25 known specimens in the world.*

*
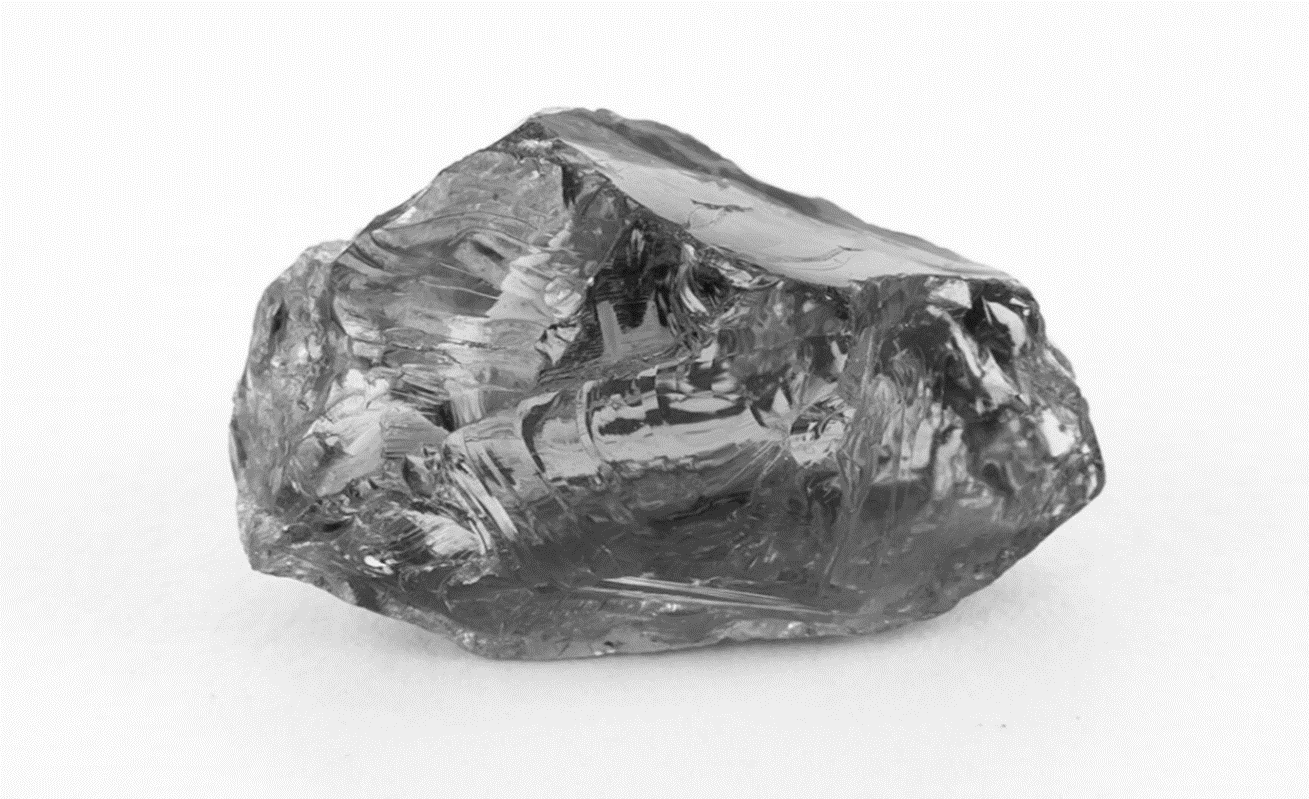
*

**Figure C. Picture of gemstone used in the study.**

Extrinsic value condition:

*Arilaete is a common type of quartz, characterised by a purplish appearance that can range from lavender to deep violet. These stones have tremendous utility value, and are used in a range of applications from watchmaking and electronics, through to esoteric practices such as crystal therapy. There are highly productive arilaete mines all over the world, which produce this gem in large quantities and massive sizes. Arilaete is also easily manufactured in the lab, making it one of the most common and readily available gemstones in the world.*

Table (see Figure D): Intrinsic value condition

*The Tarendo table, named after its creator, Thomas Tarendo, was hand-carved in the late 1700s. This antique table prefers stunning looks over functionality, with pierced fretwork, long legs ending in narrow ankles, and detailed ball-and-claw feet. Between the legs and running along the length of the table is beautiful, intricate artwork, painstakingly carved out entirely by hand. While this one-of-a-kind table wasn’t actually designed as art, its ornate construction and striking features defy us to call it anything else.*


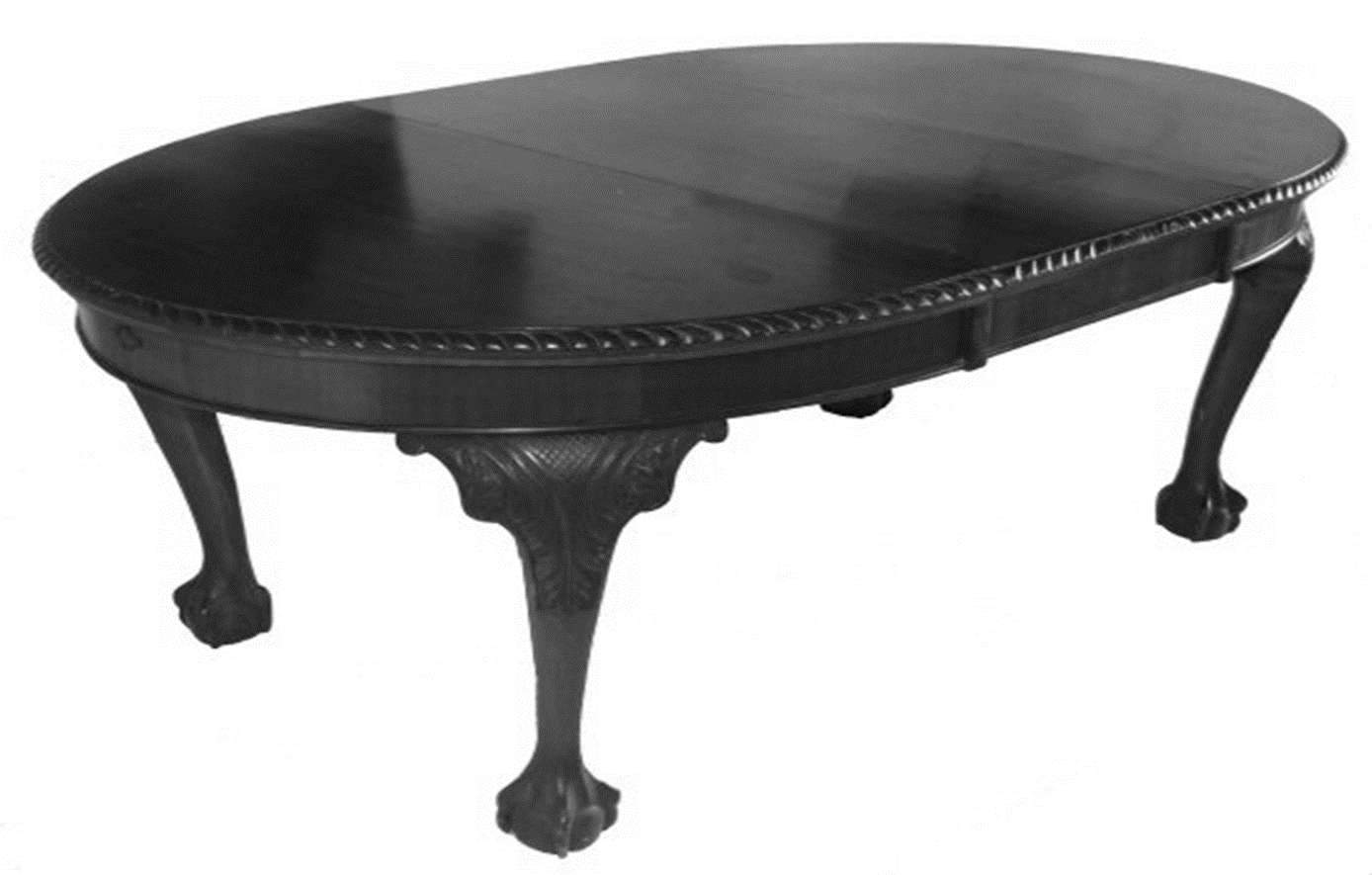


**Figure D. Picture of table used in the study.**

Extrinsic value condition

*The Tarendo is a type of table that features an oval shaped top, and short legs that end in ball-and-claw feet. Tarendo tables are renowned for their versatility. While primarily marketed as dining tables, their stylish design and neutral features make them suitable for use as coffee tables, office tables, or even as workbenches. Tarendo tables have been mass-produced by retail outlets such as Walmart, making them one of the most common tables in the western world.*

## Additional Result

For our control variables, only judgements of how harmful destruction would be to the entities’ interests were significantly higher in the intrinsic condition (intrinsic: *M* = 4.78, *SD* = 2.24; extrinsic: *M* = 4.25, *SD* = 2.40, *t*(410) = 2.31, *p* = .021); all other indicators did not significantly differ across conditions (would experience pain [intrinsic, *M* = 3.39, *SD* = 2.37 vs. extrinsic, *M* = 3.15, *SD* = 2.31], *t*(410) = 1.07, *p =* .284; matter to itself [intrinsic, *M* = 3.58, *SD* = 2.55 vs. extrinsic *M* = 3.37, *SD* = 2.45], *t*(410) = 0.85, *p* = .398). Among ratings of instrumental value only usefulness was significantly higher in the extrinsic condition (intrinsic: *M* = 4.49, *SD* = 1.82; extrinsic: *M* = 5.91, *SD* = 1.30, *t*(410) = 9.13, *p* < .001); all other indicators did not significantly differ across conditions (matter to others [intrinsic, *M* = 5.34, *SD* = 1.96 vs. extrinsic, *M* = 5.02, *SD* = 1.95], *t*(410) = 1.66, *p =* .097; economic value [intrinsic, *M* = 4.79, *SD* = 2.03 vs. extrinsic *M* = 4.86, *SD* = 1.73], *t*(410) = 0.39, *p* = .695).

In the 2-way ANOVA with intrinsic vs. extrinsic value conditions predicting judgements of wrongness to destroy and ratings associated with personhood and instrumental value entered as covariates in the model, rated extrinsic value, *F*(1,403) = 12.57, *p* <.001, economic value, *F*(1,403) = 13.21, *p* < .001, matter to others, *F*(1,403) = 86.93, *p* <.001, matter to itself, *F*(1,403) = 7.30, *p* = .007, would experience pain, *F*(1,403) = 14.38, *p* < .001, and would negatively affect its interests, *F*(1,403) = 7.76, *p* = .006, significantly predicted wrongness to destroy. Usefulness, *F*(1,403) = 2.84, *p* = .093, however, did not significantly predict wrongness to destroy.

In the 2-way ANOVA (intrinsic value vs. extrinsic value) predicting judgements of deserving punishment and ratings associated with personhood and instrumental value entered as covariates in the model, economic value, *F*(1,403) = 5.31, *p* = .022, matter to others, *F*(1,403) = 47.96, *p* < .001, matter to itself, *F*(1,403) = 5.41, *p* = .021, and would experience pain, *F*(1,403) = 10.66, *p* = .001, significantly predicted deserving punishment. Usefulness, *F*(1,403) = 3.82, *p* = .051, rated extrinsic value, *F*(1,403) = 2.98, *p* = .085, and would negatively affect its interests, *F*(1,403) = 0.28, *p* = .599, however, did not significantly predict deserving punishment.

In the 2-way ANOVA (intrinsic value vs. extrinsic value) predicting judgements of punishment severity and ratings associated with personhood and instrumental value entered as covariates in the model, economic value, *F*(1,403) = 4.83, *p* = .029, matter to others, *F*(1,403) = 18.39, *p* <.001, and would experience pain, *F*(1,403) = 31.85, *p* < .001, significantly predicted punishment severity. Usefulness, *F*(1,403)=3.48, *p*=.063, rated extrinsic value, *F*(1,403) = 1.90, *p* = .169, would negatively affect its interests, *F*(1,403) = 0.10, *p* = .747, and matter to itself, *F*(1,403) = 0.51, *p* = .476, however, did not significantly predict punishment severity.

For the regression predicting wrongness to destroy entities characterized as high in intrinsic value, matter to others, (β=.38, *p*<.001), and would negatively affect its interests (β=.14, *p*=.044) significantly predicted wrongness to destroy. Rated extrinsic value (β=-.04, *p*=.527), would experience pain (β=.13, *p*=.102), matter to itself (β=.09, *p*=.242), economic value (β=.02, *p*=.771), and usefulness (β=.10, *p*=.154) did not predict wrongness to destroy.

Furthermore, for the regression predicting deservingness of punishment, matter to others (β=.09, *p*<.256), Rated extrinsic value (β=.07, *p*=.383), would experience pain (β=.15, *p*=.166), matter to itself (β=.16, *p*=.057), would negatively affect its interests (β=-.05, *p*=.538), economic value (β=.02, *p*=.777), and usefulness (β=.09, *p*=.292) did not predict deservingness of punishment.

For the regression predicting punishment severity, would experience pain (β=.34, *p*<.001) significantly predicted punishment severity. Rated extrinsic value (β=.05, *p*=.518), matter to others (β=.14, *p*=.076), matter to itself (β=.13, *p*=.115), would negatively affect its interests (β=-.10, *p*=.206), economic value (β=.06, *p*=.368), and usefulness (β=.05, *p*=.539), however, did not predict punishment severity.

Finally, for the regression predicting costly third-party punishment, would experience pain (β=.21, *p*=.029) and matter to itself (β=.18, *p*=.037) significantly predicted costly third-party punishment. Rated extrinsic value (β=.02, *p*=.786), matter to others (β=.04, *p*=.630), would negatively affect its interests (β=-.11, *p*=.204), economic value (β=.03, *p*=.708), and usefulness (β=.01, *p*=.927), however, did not predict costly third-party punishment.

**Table A. Twenty entities presented to participants (ten intrinsic, ten low intrinsic/high means-end valuable) in Pilot Study 2.**

| **Intrinsically valuable** | **Extrinsically valuable** |
| --- | --- |
| An old growth forest  (*An old growth forest - approx. 200 years old*) | Tree plantation  (*A new pine tree plantation*) |
| Rare coin  (*A collection of rare 18^th^ century coins*) | Common currency  (*A rol1 of new bank notes*) |
| Something that is very old | A copy of something original  (*A replica of something that is very old*) |
| Culturally sacred land  (*A culturally sacred plot of land*) | Land for building development  (A plot of land for building development) |
| A diverse ecosystem | Natural resource  (*A coal mine*) |
| Historical building  (*A 16^th^ century historical building*) | A new building  (*A recently constructed building*) |
| An antique table  (*An antique table - approx. 300 years old*) | A new table |
| A family heirloom ring | A new ring |
|  |  |
| A photograph of someone’s great grandfather | A photograph of a celebrity |
| A sea turtle | Turtle meat |
| (*An original artwork by Picasso*) | (*A print of an artwork by Picasso*) |

The entities in *Italics* indicate changes and additions made in Study 1.

|  | 1I | 2I | 3I | 4I | 5I | 6I | 7I | 1E | 2E | 3E | 4E | 5E | 6E | 7E |
| --- | --- | --- | --- | --- | --- | --- | --- | --- | --- | --- | --- | --- | --- | --- |
| *Intrinsically Valuable Entities* | | | | | | | | | | | |  |  |  |
| 1I. Intrinsic Value | - |  |  |  |  |  |  |  |  |  |  |  |  |  |
| 2I. Extrinsic Value | -.117 | - |  |  |  |  |  |  |  |  |  |  |  |  |
| 3I. Experience Pain | .083 | .248^*^ | - |  |  |  |  |  |  |  |  |  |  |  |
| 4I. Harmful to interest | .136 | .242^*^ | .461^**^ | - |  |  |  |  |  |  |  |  |  |  |
| 5I. Matter to itself | .054 | .256^*^ | .882^**^ | .445^**^ | - |  |  |  |  |  |  |  |  |  |
| 6I. Matter to others | .566^**^ | .099 | -.199^*^ | .219^*^ | -.216^*^ | - |  |  |  |  |  |  |  |  |
| 7I. Wrong to destroy | .664^**^ | -.071 | .057 | .246^*^ | .026 | .705^**^ | - |  |  |  |  |  |  |  |
| *Extrinsically Valuable Entities* | | | | | | | | | | | |  |  |  |
| 1E. Intrinsic value | .267^**^ | .232^*^ | .380^**^ | .240^*^ | .374^**^ | .124 | .268^**^ | - |  |  |  |  |  |  |
| 2E. Extrinsic value | .411^**^ | .011 | -.151 | .133 | -.115 | .460^**^ | .392^**^ | .045 | - |  |  |  |  |  |
| 3E. Experience pain | -.127 | .319^***^ | .834^***^ | .349^**^ | .786^**^ | -.325^***^ | -.084 | .512^***^ | -.124 | - |  |  |  |  |
| 4E. Harmful to interest | -.042 | .254^*^ | .330^**^ | .827^***^ | .342^**^ | .130 | .123 | .376^**^ | .259^*^ | .406^***^ | - |  |  |  |
| 5E. Matter to itself | -.148 | .203^*^ | .742^***^ | .339^**^ | .846^***^ | -.339^**^ | -.129 | .483^***^ | -.085 | .878^***^ | .440^***^ | - |  |  |
| 6E. Matter to others | .143 | .175 | -.124 | .164 | -.074 | .581^**^ | .435^**^ | .371^**^ | .493^***^ | -.004 | .368^**^ | .038 | - |  |
| 7E. Wrong to destroy | .120 | .323^**^ | .315^**^ | .263^**^ | .319^**^ | .252^*^ | .439^**^ | .624^**^ | .279^**^ | .441^***^ | .468^***^ | .406^***^ | .611^***^ | - |

**Table B. Intercorrelations among all variables used in Study 1 for intrinsically valuable and extrinsically valuable entities**

^*^*p* < .05, ^**^*p* < .01, ^***^*p* < .001

**Table C. Intercorrelations between all variables used in Study 3 for intrinsically valuable and extrinsically valuable entities**

|  | 1I | | 2I | 3I | 4I | 5I | 1E | 2E | 3E | 4E | 5E |
| --- | --- | --- | --- | --- | --- | --- | --- | --- | --- | --- | --- |
| *Intrinsically Valuable Entities* |  | |  |  |  |  |  |  |  |  |  |
| 1I. Intrinsic Value | - | |  |  |  |  |  |  |  |  |  |
| 2I. Extrinsic Value | .125 | | - |  |  |  |  |  |  |  |  |
| 3I. Economic Value | .361^***^ | | .347^***^ | - |  |  |  |  |  |  |  |
| 4I. Usefulness | .068 | | .361^***^ | .398^***^ | - |  |  |  |  |  |  |
| 5I. Wrong to destroy | .468^***^ | | .158 | .316^**^ | .223^*^ | - |  |  |  |  |  |
| *Extrinsically Valuable Entities* | |  |  |  |  |  |  |  |  |  |  |
| 1E. Intrinsic value | .131 | | .284^**^ | .393^***^ | .222^*^ | .001 | - |  |  |  |  |
| 2E. Extrinsic value | .301^***^ | | .108 | .172 | .171 | .048 | .104 | - |  |  |  |
| 3E. Economic Value | .368^***^ | | .157 | .428^***^ | .120 | .400^***^ | .248^**^ | .324^**^ | - |  |  |
| 4E. Usefulness | .437^***^ | | .173 | .243^*^ | .294^**^ | .331^***^ | .145 | .496^***^ | .384^***^ | - |  |
| 5E. Wrong to destroy | .073 | | .226^*^ | .275^**^ | .246^*^ | .424^**^ | .394^***^ | .047 | .347^***^ | .333^***^ | - |

^*^*p* < .05, ^**^*p* < .01, ^***^*p* < .001

|  |  | Extrinsic Value  Emphasis | | Intrinsic Value Emphasis | |
| --- | --- | --- | --- | --- | --- |
| *Entity* | *Variable* | *M* | *SD* | *M* | *SD* |
| Cricket | Intrinsic value | 4.57 | 1.83 | 5.36 | 1.45 |
|  | Means-end value | 5.47 | 1.46 | 3.82 | 1.88 |
| Forest | Intrinsic value | 5.58 | 1.88 | 6.31 | 1.18 |
|  | Means-end value | 5.00 | 1.93 | 3.91 | 2.05 |
| Stone | Intrinsic value | 4.09 | 1.71 | 5.82 | 1.59 |
|  | Means-end value | 5.26 | 1.81 | 3.53 | 2.14 |
| Table | Intrinsic value | 3.43 | 1.68 | 5.58 | 1.20 |
|  | Means-end value | 5.47 | 1.30 | 4.00 | 1.71 |

**Table D. Means and standard deviation of the intrinsic value and means-end value variables (Study 5)**
