# Supplementary material for: Correction: The moral significance of protecting environmental and cultural objects
Source: PLoS One. 2024 Mar 22;19(3):e0300942. doi: 10.1371/journal.pone.0300942 (PMC10959328; doi:10.1371/journal.pone.0300942)
Supplement: S2 File — (PDF) [file pone.0300942.s002.pdf]

PLOS ONE

RESEARCH ARTICLE

The moral significance of protecting environmental and cultural objects

Brock Bastian[1]*, **Charlie R. Crimston**[2], **Christoph Klebl**[2], **Paul A. M. van Lange**[3]

1 Melbourne School of Psychological Sciences, Faculty of Medicine, Dentistry and Health Sciences, University of Melbourne, Parkville, Victoria, Australia, **2** School of Psychology, Faculty of Health and Behavioural Sciences, University of Queensland, Saint Lucia, Queensland, Australia, **3** Department of Experimental and Applied Psychology, Faculty of Behavioural and Movement Science, Vrije Universiteit Amsterdam, Amsterdam, The Netherlands

* brock.bastian@unimelb.edu.au

Abstract

A powerful avenue through which to promote the preservation of the natural and cultural environment is to afford cultural and environmental objects moral significance. In this research, we examine a range of factors that may give rise to moral concern regarding the protection of culturel and environmental objects as ends in themselves. In this way, we also extend theorizing and evidence beyond a focus on sentience as a focal determinant of moral significance Across five studies we show that non-sentient objects can sometimes be viewed as possessing intrinsically valuable properties that afford them moral standing (independent of their extrinsic/means-end value or any perception of their capacity to think and feel). People judge it morally wrong to harm things that are beautiful, sacred, rare, or old, and this cannot be explained merely by their usefulness or economic value. Our findings provide new insight into ways to elevate the protection of natural and cultural objects to an issue of moral significance, and suggest avenues through which to motivate the preservation of a natural and cultural environments.

OPEN ACCESS

Citation: Bastian B, Crimston CR, Klebl C, van Lange PAM (2023) The moral significance of protecting environmental and cultural objects. PLoS ONE 18(2): e0280393. https://doi.org/10.1371/journal.pone.0280393

Editor: Ali B. Mahmoud, St John's University, UNITED STATES

Received: May 28, 2021

Accepted: December 28, 2022

Published: February 9, 2023

Copyright: © 2023 Bastian et al. This is an open access article distributed under the terms of the Creative Commons Attribution License, which permits unrestricted use, distribution, and reproduction in any medium, provided the original author and source are credited.

Data Availability Statement: The data underlying these studies can be found as per the below OSF reference. Bastian, B. (2021, May 28). The moral significance of protecting environmental and cultural objects. Retrieved from: https://doi.org/10.17605/OSF.IO/FTGUE.

Funding: The research was funded by an ARC Discovery Project (DP200101446) awarded to Brock Bastian and Paul van Lange

Competing interests: The authors have declared that no competing interests exist.

Introduction

In 2001, the Taliban destroyed the Buddhas of Bamiyan, monumental statues estimated to be 1,500 years old and carved into the side of a cliff in central Afghanistan [1]. This "cultural vandalism" was met with global outrage and moral indignation [2], and such acts have more recently been subject to criminal sentencing [3]. In 2008, a referendum vote approved changes to the Ecuadorian constitution, including the granting of rights to nature [4]. A few years later, the New Zealand government granted personhood status to the Whanganui River and the 821-square-mile Te Urewera park on New Zealand's North Island, ensuring that these sites have recognized individual rights under law [5]. Such examples are consistent with the global human desire to protect cultural and natural sites enshrined in the *Convention Concerning the Protection of the World's Cultural and Natural Heritage* adopted by UNESCO in 1972. In this paper, we seek to understand the qualities of natural and environmental objects which contribute to elevating their protection as an issue of moral significance. That is, whether and how people might come to view these objects as possing direct moral standing, and thereby providing insight into avenues through which to increase engagement in conservation efforts.

What determines moral significance?

There has been a tendency for theorists to consider the interests of a sentient actor as necessary for actions, or the consequences of those actions, to have moral significance. From this perspective, what makes an action moral or immoral fundamentally depends on whether there is someone (or something) to which that action matters and who therefore has needs and preferences that deserve moral consideration. Whether it is utilitarian/consequentialist [6–8] or deontological [9] approaches to determining what is right or wrong, there ultimately needs to be an entity with 'interests' toward which moral wrongs can be directed. It was this recognition which led Aristotle to declare that there can be both moral agents–those whose actions can be morally right or wrong–and moral patients–those who can have moral right or wrong done to them (see [10]).

From this perspective, when determining whether an action is morally significant, a fundamental task is to define the basis on which someone (or something) should be viewed as a moral patient and therefore have interests that could be impacted or harmed. In the case of people, for example, this is relatively straightforward because people can think and feel–they are sentient–and therefore are capable of having interests that can be harmed. That is, they have direct moral standing. Such a task becomes harder, however, when considering those entities that are on the boundaries of what might generally be considered to be a moral patient (e.g., fetuses, animals). Such entites may often be viewed as merely possessing indirect moral standing–that is they matter to the extent that their existence or descruction matters to moral patients (those who have direct moral standing). When it comes to what determines direct moral standing, there are two basic perspectives. One is the Kantian perspective, which states that entities possess direct moral standing to the extent that they are rational. The other, referred to as the Benthamite tradition, is less restrictive and proposes that the only important consideration is whether something can suffer ([6]; see also [11]).

Psychologists have likewise begun to examine what factors give rise to perceived moral standing (for a discussion see [12]). In line with both the Kantian and Benthamite traditions, possessing mental capacities has been found to be the most important factor in determining whether people tend to view an entity as possessing direct moral standing. Most consistently, the perceived capacity for hedonic experience (i.e., to experience hunger, fear, pain and pleasure), and therefore the capacity to suffer, has been linked to the belief that people or animals should not be harmed, and that causing harm to them would be considered morally wrong [10, 13–16]. Other mental capacities, such as agency or the capacity to control and direct actions autonomously (i.e., to have self-control, memory, and planning abilities) has been found to predict whether an entity is viewed as a moral agent (capable of acting rightly or wrongly, and therefore deserving of moral praise or blame: see also [17]). Yet, in some cases agency also appears to underpin the perception that a person or animal has the right not to be harmed [15, 16] as do other dimensions such as dangerousness [15]. Critically, however, from a psychological perspective, what is generally thought to determine whether something is judged to possess direct moral standing is whether it has some level of mental capacity; that is whether it possesses a mind (see [18–20]).

Although possessing a mind appears to be centrally important for whether an entity is judged to have direct moral standing–that is, whether its interests should be respected–this psychological model of moral judgment cannot fully account for why people seek to protect environmental and cultural objects (which don't have minds) and become outraged when they are destroyed.

A broader framework for moral standing

One reason that people might want to protect cultural and environmental objects or sites is because they provide for their own (human) benefits. Rivers, forests, and historical artifacts

contribute to human welfare because they provide for enjoyment, water and clean air, and may also be symbolic of group identity (e.g., [21–24]). Yet, these apparent instrumental motivations stand in stark contrast with our reasons for protecting humans. The human rights movement arose from an appreciation that humans possess direct moral standing–it is an individual's *own* interests and not the interests of *others* that affords him or her the right to personal protection [25, 26]. The same rationale is also evident within the animal rights movement, where the perceived ability for animals to think and feel, and therefore their capacity to have their own (at least minimal) interests, is viewed as a basis for their direct moral standing (see [11, 27]), and makes their protection an issue of moral significance.

Current psychological frameworks of moral judgement fall short in providing a basis on which cultural, environmental, and other non-sentient objects may be considered worthy of protection for reasons beyond the mere satisfaction of human needs and preferences. This leaves little theoretical ground for understanding recent decisions to protect parks and rivers in New Zealand, the UNESCO convention, or the current trend towards expanding our limits of moral consideration [25–29]. Indeed, research suggests that people frequently see non-sentient objects as deserving of direct moral standing, placing them within the boundaries of their moral consideration (e.g., the environment, plants, low-sentient animals; [30]). It is for this reason that philosophers have attempted to establish a broader basis for direct moral standing (e.g., [31–36]; see also [37]).

In order to progress an account of the psychology behind broader conceptions of direct moral standing, we draw on the concept of *intrinsic* value (see [38] for a similar approach). This type of value can be contrasted with instrumental or extrinsic value and focuses on whether something is seen as valuable in its own right, and not because of how it might be useful for other ends. Here we use the term with specific reference to the intrinsically valuable properties of an entity which afford it respect and consideration, and as such, contriute to it being judged as possessing direct moral standing (see [39]; see also work on final value–[40]). By drawing on this broader conception of value, we aim to develop a framework for considering which properties of cultural or environmental objects ground direct moral standing (or moral considerability, [32]; see Fig 1). Specifically, we ask the question of (a) whether there are qualities of environmental and cultural objects which motivate people to protect them for reasons other than their usefulness or as means to another end, and (b) whether those qualities

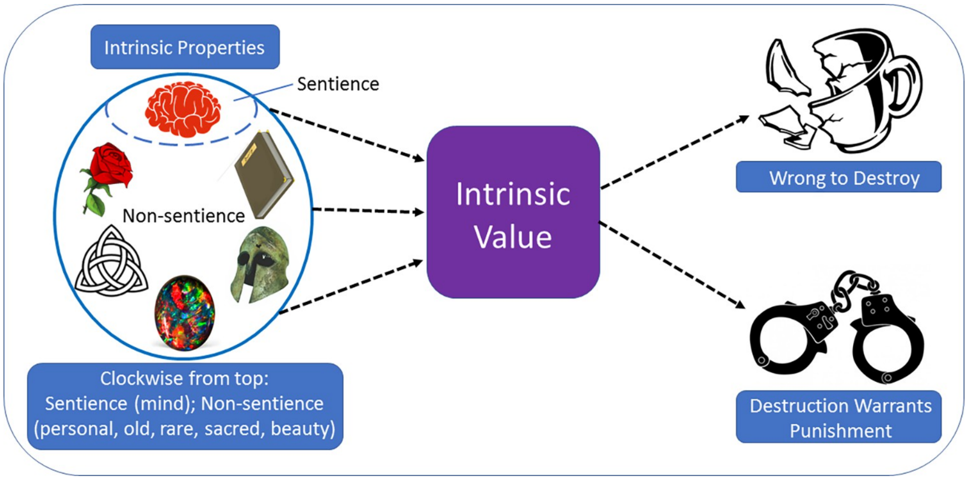

Fig 1. Pictorial representation of the relationship between intrinsic properties, attributions of intrinsic value, and moral decision making.

https://doi.org/10.1371/journal.pone.0280393.g001

extend beyond the possession of mental capacites (i.e., the capacity to have needs and preferences), but which elicit a similar moral motivation towards protecting environmental and cultural objects from harm.

Present studies

In order to gain insight into the types of entities and attributes that people view as possessing high intrinsic value (having value in their own right) and low intrinsic value (having value as a means to another end) we conducted two pilot studies. In Pilot 1, participants were provided with descriptions of intrinsic and extrinsic value and asked to generate separate lists of entities they considered to be representative of each type of value. In Pilot 2, we confirmed the representativeness of each list by asking another group of participants to rate the exemplars on the same descriptions of intrinsic and extrinsic value (see S1 File). In Pilot 2, we also asked participants to rate a list of 14 attributes that we believed captured the qualities which afford certain entities high vs. low levels of intrinsic value (e.g., old vs. new, unique vs. common; see S1 File).

Based on these initial observations, in Studies 1–3 we first investigated whether people do find it more morally wrong to destroy objects that have high (vs. low) intrinsic value, independent of their means-end value and in a way that is not limited to (and therefore goes beyond) any perception of their capacity to think and feel. Our aim in these first three studies was to provide a framework for considering a broader range of attributes which might elevate the motivation to protect an entity from harm. In Studies 4–5, drawing on our pilot data, we turned attention to an analysis of the specific kinds of qualities that afford entities high levels of intrinsic value, and therefore elevate their protection to the status of a moral issue.

Our designs and analyses were guided by several methodological principles. One principle is that we wanted to capture entities that are part of people's lives, and therefore our pilot studies focused on participant-generated entities that are considered to possess high vs. low intrinsic value. A second principle is that across the five studies we focus on the unique contribution of intrinsic value above and beyond extrinsic or means-end value (such as economic value and usefulness) and/or sentience in predicting moral evaluations, as well as behavioral responses; in particular, punishment. For this reason, we aimed to compare entities considered to be high in intrinsic value to entities considered to be high in extrinsic or means-end value. Although these two forms of value may be interdependent, here we drew on descriptions of intrinsic and extrinsic value which focused both on the existence of each type of value (e.g., intrinsic: valued for its own sake vs. extrinsic: is an effective means to an end) and also sought to distinguish these two types of value (e.g., intrinsic: are not valued for the sake of something else vs. extrinsic: are not valued for their own sake). Teasing apart these two types of value was important for our purposes, such that we could compare how each type plays into judgements of direct moral standing. In this way, we aimed to provide insight into specific attributes which may be emphasized (and which extend beyond sentience) in order to elevate the protection of environmental and cultural objects to an issue of moral significance.

Study 1

In Study 1, we examined whether people judge the destruction of intrinsically valued entities as more morally wrong than the destruction of extrinsically valued entities. In order to generate lists of entities that are considered to represent high intrinsic value vs. high extrinsic value, we conducted two pilot studies (see S1 File for full reporting). In both pilot studies we provided participants with a description of intrinsic and extrinsic value, again noting that our approach was to distinguish these two froms of value (we note that in later studies we used a sparser definition of these two forms of value–see examples provided below). In Pilot Study 1 participants

Table 1. Twenty-two entities presented to participants in Studies 1, 2, and 3.

Intrinsically valuable	Extrinsically valuable
An old growth forest—approx. 200 years old	A new pine tree plantation
A collection of rare 18th century coins	A rol1 of new bank notes
Something that is very old	A replica of something that is very old
A culturally sacred plot of land	A plot of land for building development
A diverse ecosystem	A coal mine
A 16th century historical building	A recently constructed building
An antique table—approx. 300 years old	A new table
A family heirloom ring	A new ring
A photograph of someone's great grandfather	A photograph of a celebrity
A sea turtle	Turtle meat
An original artwork by Picasso	A print of an artwork by Picasso

https://doi.org/10.1371/journal.pone.0280393.t001

were required to generate a list of things that were primarily valuable to them because of their intrinsic or extrinsic value. From these responses we generated a list of objects that were commonly considered to possess either intrinsic or extrinsic value. In developing our list, we attempted to match objects across both types of value. For instance, an original artwork (intrinsic value), a copy of an artwork (extrinsic value), a sea turtle (intrinsic value), sea turtle meat (extrinsic value). In Pilot Study 2 we aimed to validate this list by asking participants to rate each of the objects on the extent to which they possessed intrinsic or extrinsic value, again drawing on the definitions provided. This revealed that the intrinsically valuable entities were rated as higher on intrinsic value than the extrinsically valuable entities, and vice versa. These lists were then drawn on in Study 1 to determine which entities (intrinsically vs. extrinsically valuable) were viewed as more wrong to destroy (see Table 1). Wrongness to destroy was our primary dependent variable and ratings of intrinsic value was our independent variable. In order to show that ratings of intrinsic value predicted unique variance in judgements of wrongness to destroy, beyond any variance associated with non-intrinsic sources of value (i.e., how much destroying the entity might matter to others, ratings of extrinsic value) or the possession of mental capacities or interests (i.e., would the entity experience pain, does it have interests that are negatively impacted) we included a number of control variables in our analyses.

Method

All studies originated in Australia and received clearance from the Human Research Ethics Committee (#HC14343). Written consent was obtained in accordance with ethical requirements. For all studies we report all measures, conditions, data exclusions, and our methods for determining sample sizes. Data are available at https://doi.org/10.17605/OSF.IO/FTGUE

Participants

We determined that a sample size of 95 would allow for an 80% chance of observing medium-sized effects as significant at the 5% level. Ninety-eight participants (56 male, *Mage* = 36.46, *SD* = 11.96, 95 native English speakers) from Amazon's Mechanical Turk (MTurk) completed the survey online in exchange for a small fee.

Materials

Participants rated a list of 22 exemplars (11 high in intrinsic value and 11 high in extrinsic value with items paired across lists; e.g., an old-growth forest vs. a tree plantation; an antique

table vs. a new table) closely based on the pilot lists (see Table 1). Participants first read definitions of intrinsic and extrinsic value and then rated each of the exemplars (one at a time in random order) on seven questions in the order presented below. Twenty-two objects were presented to participants in total.

Moral significance

For our key dependent variable participants rated "How wrong would it be to destroy this entity?" on seven-point Likert-type scale (1 = *not at all*, to 7 = *very wrong/very much so*).

Control variables

Mental capacity/Sentience. We also asked whether participants felt that destroying the entity would influence other mental-capacity or sentience-related considerations ("Would destroying this entity negatively affect its interests?"; "To whom would it matter if this entity was destroyed—would it matter to the entity itself?"; "How much pain or suffering would this entity experience if it were destroyed"). Participants responded to all questions by clicking on a seven-point Likert-type scale (1 = *not at all*, to 7 = *very wrong/very much so*). The purpose of these measures was to determine whether ratings of intrinsic value explained unique variance in ratings of moral wrongness to destroy, beyond any variance explained by perceived mental capacity or sentience. While our theoretical formulation acknowledges that mental capacity is an important source of intrinsic value, our aim was to show mental capacity alone could not account for the variance explained by judgements of intrinsic value.

Non-intrinsic sources of value. We included a question designed to capture the extent to which destruction of an entity would matter for reasons unrelated to the intrinsic value of the entity, but rather to how its destruction might impact on the welfare of others ("To whom would it matter if this entity was destroyed–would it matter to other people?"). Participants responded by clicking on a seven-point Likert-type scale (1 = *not at all*, to 7 = *very wrong/very much so*).

Value type

Participants rated how much intrinsic value the entity possessed ("How much does this entity possess intrinsic value; i.e., value in its own right?") and how much extrinsic value the entity possessed ("How much does this entity possess extrinsic value; i.e., is it valued as an effective means to an end?") on seven-point Likert-type scale (1 = *not at all*, to 7 = *very wrong/very much so*). Ratings of intrinsic value were treated as our key independent variable.

Results and discussion

For each measure, we combined the ratings of intrinsic and extrinsic entities, respectively (alphas reported below). We first examined whether the set of exemplars represented intrinsic and extrinsic value, respectively, finding that participants attributed more intrinsic value ($M = 5.58$, $SD = 1.27$, $\alpha = .90$) than extrinsic value ($M = 3.38$, $SD = 1.47$, $\alpha = .90$) to intrinsic exemplars, $t(97) = 8.40$, $p < .001$, 95% CI [1.33,2.15], whereas they judged the extrinsic exemplars as higher in extrinsic value ($M = 4.57$, $SD = 1.25$, $\alpha = .85$) than in intrinsic value ($M = 2.98$, $SD = 1.05$, $\alpha = .78$), $t(97) = 9.90$, $p < .001$, 95% CI [1.27,1.91].

Turning to our key dependent variable, participants reported that it would be more wrong to destroy the intrinsic exemplars ($M = 5.79$, $SD = 1.09$, $\alpha = .91$) than the extrinsic exemplars ($M = 3.64$, $SD = .95$, $\alpha = .83$), $t(97) = 19.74$, $p <. 001$, $d = 1.87$, 95% CI [1.93, 2.37]. Paired-

sample t-tests revealed that, for each entity on the list, participants deemed destroying something with intrinsic value to be more wrong than destroying its extrinsically valuable counterpart (all p values $< .001$).

Employing this same approach, analysis of the control variables revealed that participants afforded more mental capacity or interests to the intrinsic exemplars (entity would experience pain if destroyed [intrinsic, $M = 2.73$, $SD = 1.19$, $\alpha = .86$ vs. extrinsic, $M = 1.77$, $SD = .94$, $\alpha = .88$], $t(97) = 14.51$, $p < .001$; destruction would negatively affect the entity's interests [intrinsic, $M = 4.03$, $SD = 1.62$, $\alpha = .92$ vs. extrinsic, $M = 3.12$, $SD = 1.35$, $\alpha = .90$], $t(97) = 10.24$, $p < .001$; and destruction would matter to the entity itself [intrinsic, $M = 2.74$, $SD = 1.12$, $\alpha = .85$ vs. extrinsic $M = 1.74$, $SD = .99$, $\alpha = .88$], $t(97) = 16.18$, $p < .001$). Participants also thought it would matter more to other people if intrinsic exemplars were destroyed (would matter to other people [intrinsic, $M = 6.12$, $SD = .84$, $\alpha = .88$ vs. extrinsic, $M = 4.70$, $SD = 1.15$, $\alpha = .84$], $t(97) = 14.65$, $p < .001$) however, participants afforded greater extrinsic value to extrinsically valuable exemplars [intrinsic, $M = 3.84$, $SD = 1.46$, $\alpha = .90$ vs. extrinsic, $M = 4.57$, $SD = 1.25$, $\alpha = .85$], $t(97) = 3.74$, $p < .001$).

To provide further support to our t-test comparisons of our main dependent variable, wrongness to destroy, we ran a mixed-model ANOVA with judgements of wrongness to destroy intrinsic vs. extrinsic valuable entities as the within-participant variable, and difference scores between the mean ratings of intrinsic exemplars and the mean ratings of extrinsic exemplars for all ratings associated with mental capacity/sentience and non-intrinsic sources of value entered as covariates in the model. This revealed that within-subject differences in moral wrongness remained significant while controlling for these covariates, $F(1,92) = 21.91$, $p < .001$, $\eta_p^2 = .19$.

Finally, having determined that the intrinsic exemplars were viewed as possessing higher levels of intrinsic value and as more wrong to destroy, we next focused specifically on the set of eleven intrinsic exemplars and examined which judgements predicted wrongness to destroy. Regression analysis tested the relationship between judgments of intrinsic value and moral wrongness of destruction controlling for other non-intrinsic sources of value. In order to best estimate the averaged correlations, we created z-scores for each variable within each entity prior to averaging those variables across entities. We employed this same procedure in all other averaged correlational and regression analyses in the paper.

The overall model was significant, $F(6, 91) = 26.20$, $p < .001$, $R^2 = .63$), with concerns about whether an entity's destruction would impact on the welfare of others, $\beta = .57$, $p < .001$, and its possession of intrinsic value, $\beta = .31$, $p < .001$, the only significant predictors. Ratings related to attribution of mental capacities or sentience were non-significant predictors (pain, $\beta = .13$, $p = .350$; matter to itself, $\beta = .04$, $p = .785$, harmful to interests, $\beta = .04$, $p = .472$) and whether the entity possessed extrinsic value was a significant, but negative predictor of wrongness, $\beta = -.14$, $p = .045$.

This analysis reveals that although previous research has shown possessing mental capacities and sentience contributes (perhaps most strongly in the case of sentient beings) to judgments of the wrongness of destroying an entity, there is an important, residual aspect of non-sentience-related intrinsic value that extends beyond these attributes alone. Furthermore, the unique variance in judgements of wrongness explained by ratings of intrinsic value cannot be accounted for by non-intrinsic sources of value, such as concerns regarding how much an entity's destruction might negatively impact the welfare of others. Consistent with our argument, this suggests that other properties (unrelated to sentience or mental capacity but nevertheless contributing to judgments of intrinsic value) may also give rise to perceived moral standing.

Study 2

Our findings in Study 1 revealed that people believe it is more morally wrong to destroy intrinsically valuable exemplars than extrinsically valuable exemplars. The relationship between moral wrongness and judgments of intrinsic value was not reducible to factors pertaining to sentience or mental capacity, and not accounted for by other non-intrinsic sources of value. Nonetheless, concerns over whether destroying an entity might negatively impact on the welfare of others was the strongest predictor. It is important to note that controlling for this variable provides a conservative test of our hypothesis, as whether an entity's destruction matters to others may be due in part to its high intrinsic value for them (e.g., a family heirloom ring). Nonetheless, our aim is to demonstrate that people's judgments of the moral wrongness of destruction are not exclusively tied to their concerns for other sentient beings (i.e., its indirect moral standing). Therefore, in Study 2 we set out to appropriately control for this alternative non-intrinsic source of value by drawing on a thought experiment used in the field of environmental ethics referred to as 'the last man argument' [35]. This has been used to show that people often feel it is wrong to destroy environmental entities, even though there are no persons left to whom their existence would matter (i.e., due to the entities direct moral standing). Our aim was to show that under these conditions, people would judge the destruction of intrinsically valuable entities as more morally wrong than the destruction of extrinsically valuable entities.

Method

Participants. Based on the effect sizes of the main analyses in Study 1 (d = 1.87 and η_p^2 = .19), we determined requiring a sample of N = 18 to allow for an 80% chance of observing this sized effect as significant at the 5% level [41]. We recruited a larger sample of 102 participants (75 male, *Mage* = 31.42, *SD* = 11.13, 98 native English speakers) from MTurk who completed the survey online in exchange for a small fee.

Materials. To begin, participants were asked to imagine the following scenario (taken from [35]):

> *"Imagine the entire world has collapsed and there is just one man left. He is about to depart the earth for another planet. Before he goes, he decides to destroy many things that are left on earth as there are no humans left."*

This scenario asks participants to imagine the value of particular entities in a context where there are no 'persons' left to whom those entities might matter. Participants were then immediately presented with the list of twenty-two entities used in Study 1 (see Table 1; the list comprises 11 matched pairs). For each entity, they were asked "How wrong do you think it would be if the last person destroyed this entity?" (1 = *not at all wrong*, to 7 = *very wrong*).

Results and discussion. To examine whether participants felt it would be more wrong for the last man standing to destroy the exemplars high on intrinsic value vs. extrinsic value we compared average judgments regarding each type of exemplar. As predicted, participants reported that it would be more wrong to destroy the intrinsic exemplars (M = 4.64, SD = 1.61, α = .93) than to destroy the extrinsic exemplars (M = 3.06, SD = 1.69, α = .94), $t(101) = 12.17$, $p < .001$ d = 1.23, 95% CI [1.32, 1.84]. Paired-sample t-tests revealed that, for each entity on the list, participants deemed destroying something with high-intrinsic value to be more wrong than destroying its high extrinsically valuable counterpart (all p values $<$ .001).

Study 3

One further possibility not addressed in Studies 1 and 2 is that it may be economic value or the usefulness of each entity which predicts whether it is morally wrong to destroy it. We addressed this in Study 3.

Method

Participants. Based on the effect size in Study 2 (d = 1.23), we determined that a sample of $N = 24$ would allow an 80% chance of observing this sized effect ($\alpha = .05$; Faul et al., 2009). We recruited 109 participants (45 male, *Mage* = 34.50, *SD* = 11.12, 108 native English speakers) from MTurk who completed the survey online in exchange for a small fee.

Materials. Participants were presented with the list of twenty-two entities used in the previous studies in random order (see Table 1). Similar to Study 1 they answered five questions about each entity in the order presented below.

Value type. Participants responded to the same items regarding the intrinsic and extrinsic value of each entity, as in Study 1.

Control variables. *Economic value and usefulness*. Participants were asked to indicate "To what extent are the following entities/items economically valuable?" and "To what extent are the following entities/items useful?" Participants responded to all questions about each of the entities presented on a scale from 1 = *not at all wrong*, to 7 = *very wrong*.

Moral significance. Participants responded to the same item regarding how wrong it would be to destroy the entity, as in Study 1.

Results and discussion. To determine that both sets of exemplars represented high intrinsic and high extrinsic value items, respectively, we compared ratings of intrinsic value and extrinsic value within each set. This revealed that, as before, the intrinsic exemplars were judged as possessing more intrinsic value ($M = 5.10$, $SD = 1.13$, $\alpha = .84$) than extrinsic value ($M = 4.36$, $SD = 1.21$, $\alpha = .85$), $t(108) = 4.98$, $p < .001$. So too, the extrinsic exemplars were judged as possessing more extrinsic value ($M = 4.08$, $SD = 0.93$, $\alpha = .72$) than intrinsic value ($M = 3.64$, $SD = 1.01$, $\alpha = .76$), $t(108) = 3.54$, $p = .001$.

Next, we compared average judgments regarding exemplars high on intrinsic value vs. high on extrinsic value. Participants again reported that it would be more wrong to destroy the intrinsic exemplars ($M = 5.90$, $SD = 1.05$, $\alpha = .91$) than to destroy the extrinsic exemplars ($M = 4.26$, $SD = 1.14$, $\alpha = .84$), $t(108) = 14.53$, $p < .001$, $d = 1.45$, 95% CI [1.41, 1.86]. Paired-sample *t*-tests revealed that, for each entity on the list, participants deemed destroying something with high-intrinsic value to be more wrong than destroying its extrinsically valuable counterpart (all p values $< .001$).

When considering the control variables, we found that participants rated the intrinsic exemplars as similar in economic value ($M = 4.52$, $SD = 1.02$, $\alpha = .80$) to the extrinsic exemplars ($M = 4.43$, $SD = 0.79$, $\alpha = .67$), $t(108) = 1.00$, $p = .318$, but rated the extrinsic exemplars as more useful ($M = 4.40$, $SD = 0.75$, $\alpha = .68$) compared to the intrinsic exemplars ($M = 4.03$, $SD = 1.06$, $\alpha = .84$), $t(108) = 3.51$, $p = .001$.

To provide further support to our *t*-test comparisons of our main dependent variable wrongness to destroy, we ran a mixed-model ANOVA with judgements of wrongness to destroy intrinsic vs. extrinsic valuable entities as the within-subjects factor, and difference scores between the mean ratings of intrinsic exemplars and the mean ratings of extrinsic exemplars for all ratings associated with instrumental value entered as covariates in the model. This revealed that the within-subject difference in moral wrongness remained significant, $F(1,105) = 88.00$, $p < .001$.

As in Study 1, having determined that the intrinsic exemplars were viewed as possessing higher levels of intrinsic value and as more wrong to destroy, we next focused specifically on the set of 11 intrinsic exemplars and examined which judgements predicted wrongness to destroy. We computed z-scores for all raw responses prior to averaging them across entities. Regression analysis tested the relationship between judgments of intrinsic value and moral wrongness of destruction controlling for other non-intrinsic sources of value. The overall model was significant, $F(4,104) = 9.59$, $p < .001$, $R^2 = .27$. Only judgments of intrinsic value, $\beta = .41$, $p < .001$, were a significant predictor (economic value, $\beta = .11$, $p = .269$; usefulness, $\beta = .16$, $p = .089$; rated extrinsic value, $\beta = .04$, $p = .965$).

Study 3 replicated the findings of Studies 1 and 2, showing that intrinsic value consistently predicted the wrongness of destroying an entity. It also went further to show that other sources of non-intrinsic value, such as economic value or usefulness, cannot account for this relationship.

Study 4

Studies 1, 2, and 3 provide evidence that some environmental and cultural objects are considered to possess intrinsic value, and to the extent they are, causing harm to them is also viewed as more morally wrong. Furthermore, this source of value is not reducible to properties associated with mental capacity or sentience, suggesting that other attributes are playing a role. This finding is consistent with broader conceptions of moral standing (or moral considerability) within the literature on environmental ethics (e.g., [31–36]). In Study 4, we aimed to advance our analysis using a larger set of exemplars with the aim of understanding what properties of non-sentient entities give rise to perceived intrinsic value. To test this, we first drew on additional data from Pilot Study 2 (see S1 File for full reporting). In that study, participants were asked to rate a list of attributes on the extent to which each would likely be used to describe an object that holds intrinsic vs. extrinsic value. Based on these ratings, we developed a list of six intrinsically valuable properties and their low intrinsic value opposites (*beautiful* vs. *ugly*, *rare* vs. *common*, *old* vs. *new*, *personal* vs. *public*, *sacred* vs. *secular*, and *possessing mind* vs. *not possessing mind*). This list was used in Pilot Study 3 (see S1 File for full reporting), in which participants were asked to generate examples of things/objects/entities that are known for possessing each of the specific properties. Specifically, they were provided with the list of six intrinsically valuable properties and their low intrinsic value opposites and asked to write down several entities that possessed each property–e.g., to provide examples of things that are considered *beautiful* as well as things that are considered *ugly*. From this task, we established a list of 60 entities to use in Study 4 (five entities representative of each of the 12 properties listed above; see Table 2).

Method

Participants. We recruited 108 participants (58 male, *Mage* = 35.94, *SD* = 10.82, 105 native English speakers) from MTurk who completed the survey online in exchange for a small fee.

Materials. Participants were presented with the list of 60 entities detailed above. They were asked to respond to 12 questions relating to all 60 entities. Ratings were provided across all entities addressing each question in turn, and in the order presented below.

Value type. As in Study 1, participants rated each entity on the extent to which it possessed intrinsic value or extrinsic value using a 7-point scale (0 = *not at all*, to 6 = *very much so*). We updated our definitions of intrinsic vs. extrinsic value, so as to focus more precisely on just these concepts. Our original definitions (see Pilot 1 in S1 File) had included statements

Table 2. Sixty exemplars selected to represent the twelve characteristics associated with high vs. low intrinsic value (Study 4).

High Intrinsically Valuable Entities					
**Beautiful**	**Personal**	**Rare**	**Sacred**	**Old**	**Possess Mind**
Rainforest	Personal diary	Historical document	Church	Antique cupboard	People
Mountain meadow	Wedding ring	Rare baseball card	Bible	Ancient ruins	Dog
Waterfall	Family photo	Rare record	Crucifix	Historic building	Dolphin
Piece of art	Personal photo	Endangered animal	Cemetery	Antique car	Monkey
Rose	Family heirloom	Rare coin	Mosque	Ancient art work	Pig
Low Intrinsically Valuable Entities					
**Ugly**	**Public**	**Common**	**Secular**	**New**	**No-Mind**
Rubbish	Park	Car	School	iPhone	Robot
Wasteland	Library	Rock	Government building	New car	Digital clock
Highway	Train station	Telephone	Football stadium	Apple watch	Table
Weed	Sidewalk	Television	Court house	3D printer	Vegetable
Abandoned car	Town square	Coin	Post office	Rookie baseball card	Computer

https://doi.org/10.1371/journal.pone.0280393.t002

that referenced personal value and replicability, which represent offshoots of something's being intrinsically valuable, rather than the core concept itself (see [42]). The updated definitions were as follows:

Intrinsic value: *Some things have value in their own right. They are not valuable because they can do something for us or provide something for us; instead, they are simply valuable for their own sake. This kind of value is often referred to as intrinsic value. In essence, intrinsically valuable things (or entities) are valuable for their own sake, and not for the sake of something else.*

Extrinsic value: *Some things have value because they provide an effective means to an end. They are not valuable for their own sake; instead, they are valuable because they can do something for us or provide something for us. This kind of value is referred to as extrinsic value (or instrumental value). In essence, extrinsically valuable things [or entities] are valuable not for their own sake, but for the sake of something else.*

Intrinsically valuable properties. In order to determine whether specific properties were associated within increased intrinsic value, and in turn, moral significance, participants rated each entity on how much it was *beautiful*, *rare*, *old*, *personal(sentimental)*, *sacred*, *or* possessed *mind and mental capacities* using a 7-point scale (0 = *not at all*, to 6 = *very much so*).

Control variables. *Economic value and usefulness*. To capture other sources of non-intrinsic value, participants also responded to the same items from Study 3 designed to capture other sources of non-intrinsic value (*economic value*, and *usefulness*) using a 7-point scale (0 = *not at all wrong*, to 6 = *very wrong*).

Moral significance. Participants responded to the same item regarding how wrong it would be to destroy the entity, as in Study 1 (0 = *not at all wrong*, to 6 = *very wrong*). Additionally, they also rated how much money, at minimum, it would take for them to destroy each entity (10-point scale; 1 = *$100*, 2 = *$500*, 3 = *$1000*, 4 = *$5000*, 5 = *$10*,000, 6 = *$50,000*, 7 = *$100,000*, 8 = *$500,000*, 9 = *$1 Million*, 10 = *I would never do it no matter how much I was paid*). This was modelled on a similar measure used by Tetlock, Kristel, Elson, Green, & Lerner [43] and was treated as an additional dependent variable.

Results and discussion. We averaged across participant responses so that each entity had an overall mean rating for each judgment. We then performed bivariate by-item correlations and regressions to explore relationships among these ratings at the item level. As shown in Table 3, the perceived intrinsic value of each entity was significantly related to judgments of "How wrong would it be to destroy this entity?", and "How much money would you require to

Table 3. Correlations between average ratings for Study 4.

	Intrinsic value	Rare	Old	Personal	Mind/mental capacities	Sacred	Beautiful	Extrinsic value	Useful	Economic value	How wrong to destroy?
Intrinsic Qualities											
Rare	.654**										
Old	.526**	.710**									
Personal	.483**	.144	-.026								
Mind/Mental Capacities	.325*	-.020	-.230	.060							
Sacred	.689**	.390**	.547**	.477**	.080						
Beautiful	.837**	.352**	.379**	.334**	.273	.558**					
Extrinsic Qualities											
Extrinsic value	-.206	-.250	-.320*	.065	-.117	-.186	-.128				
Useful	-.363**	-.452**	-.434**	-.089	.008	-.318*	-.220	.930**			
Economic value	.036	.321*	.039	-.026	-.124	-.177	-.015	.639**	.460**		
Dependent Variables											
How wrong to destroy?	.764**	.422**	.486**	.167	.460**	.647**	.688**	.014	-.034	.054	
How much money to destroy?	.603**	.348**	.453**	.012	.416**	.500**	.582**	.077	.056	.135	.956**

*$p < .05$
**$p < .01$

https://doi.org/10.1371/journal.pone.0280393.t003

destroy this entity?". Furthermore, five of the six intrinsically valuable properties showed similar relationships. In contrast, none of the judgments related to non-intrinsic value (rated extrinsic value, economic, useful) were correlated with moral judgments of wrongness or the personal economic threshold for destroying a given entity. This demonstrated that non-sentient properties of objects, such as beauty or rarity, are related to whether it is considered morally wrong to destroy those objects.

Our analysis suggests several qualities that may afford entities high levels of perceived intrinsic value, and in turn elevate their protection as an issue of moral significance. Specifically, we argue that attributes such as beauty, rarity, sentimentality (personal), sacredness, age, or mental capacity have the capacity to trigger the perception that an entity possesses intrinsic value, and in turn, moral standing. To put this more concretely, we aimed to examine whether the intrinsically valuable properties (beautiful, rare, old, personal(sentimental), sacred, possessing mind and mental capacities) impacted on judgements of wrongness or the personal economic threshold for destroying a given entity through perceptions of intrinsic value. To this end, a series of indirect effect analyses using Hayes [44, 45] PROCESS model 4 were conducted to test the prediction that the perception of beauty, rarity, personal(sentimentality), sacredness, age, or mental capacity would trigger a perception of an entity as possessing intrinsic value, and in turn, direct moral standing. As shown in Fig A and B in S1 File, results from 95% confidence intervals (with 5000 resamples) revealed significant indirect effects of each of the specific properties on both "How wrong would it be to destroy. . ." (see Fig 2), and "How much money would you require to destroy. . ." (see Fig 3), through the perception of intrinsic value.

Study 5

Studies 1–4 demonstrate that judgments of intrinsic value provide a reliable indicator of direct moral standing–that is, they predict judgments of wrongness to destroy controlling for sources

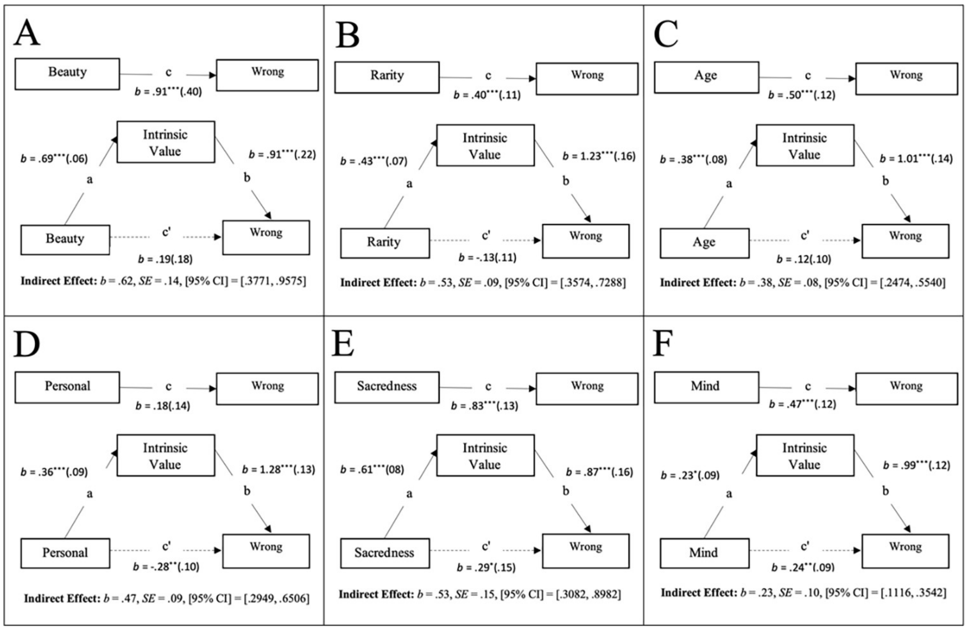

Fig 2. Mediation models for wrongness to destroys Study 4. Notes: Unstandardized coefficients and standard errors (in Study 4) for effects of Beauty (Panel A), Rarity (Panel B), Age (Panel C), Personal (Panel D), Sacredness (Panel E) and Mind Possession (Panel F), on "How wrong would it be to destroy. . .", via the Mediator of Intrinsic Value. In each panel, the top model represents the total effect, and the bottom model represents the direct/indirect effects.

https://doi.org/10.1371/journal.pone.0280393.g002

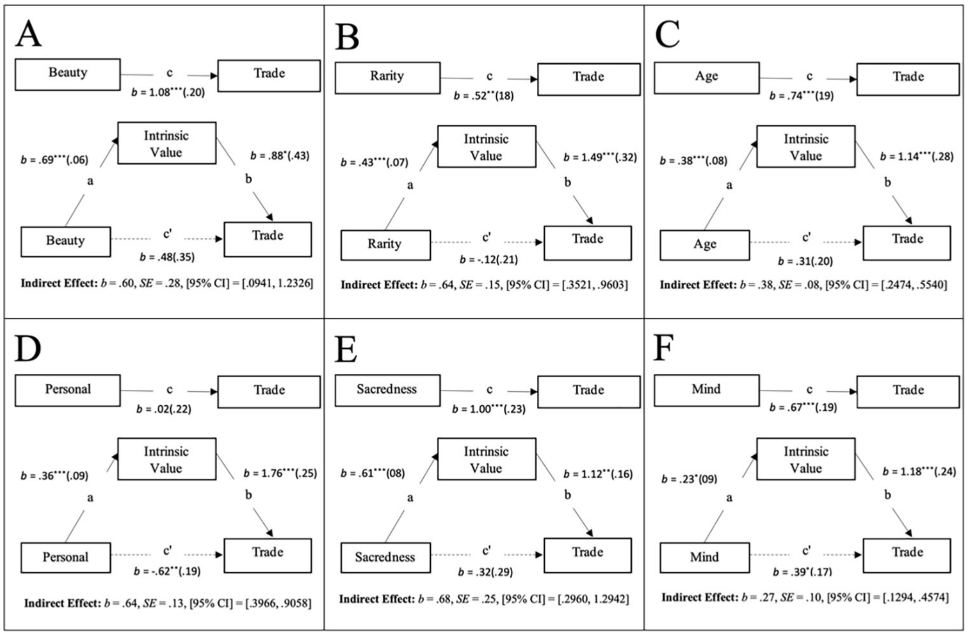

Fig 3. Mediation models for value tradeoffs Study 4. Notes: Unstandardized Coefficients and Standard Errors (in Study 4) for effects of Beauty (Panel A), Rarity (Panel B), Age (Panel C), Personal (Panel D), Sacredness (Panel E) and Mind Possession (Panel F), on "How much money it would take for them to destroy each entity . . .", via the Mediator of Intrinsic Value. In each panel, the top model represents the total effect, and the bottom model represents the direct/ indirect effects.

https://doi.org/10.1371/journal.pone.0280393.g003

of non-intrinsic value (i.e., contributing to an entities indirect moral standing). Furthermore, they show that a broad range of attributes may contribute to perceptions of high intrinsic value. These attributes include mental capacity/sentience, but also extend to a range of non-sentient properties (e.g., beauty, rarity, age, sentimentality, sacredness). In Study 5, we aimed to extend these findings in important respects. First, although we used lists of matched entities (intrinsic vs. extrinsic value) thus far, we have not shown that emphasizing attributes which afford high-intrinsic vs. high extrinsic value to an entity can shift its perceived moral standing. Second, we wanted to examine whether people might be more inclined to recommend harsh punishment or engage in acts of costly punishment for those who destroy entities high in intrinsic value.

Method

Participants. We determined that a sample size of 400 would allow for an 80% chance of observing small sized effects as significant at the 5% level. We recruited 412 participants (218 males, mean age = 34.72 years) from MTurk who completed the survey online in exchange for a small fee.

Materials. Participants were presented with a picture of one of four entities: an old growth forest, a cricket, a gemstone, or a table. Under each image was a short description, which emphasized properties shown in Study 4 to afford high-levels of intrinsic value (i.e., emphasizing beauty, rarity, age, or mental capacity) or high extrinsic value (i.e., emphasizing economic value, usefulness; see S1 File for full details). The experiment was a 4(entity type) x 2(value emphasis) fully between-subjects design, with each participant only rating one object and only reading one value emphasis. Participants responded to twelve questions in the order presented below.

Value type. Participants were next provided with the definitions of intrinsic and extrinsic value (as in Study 4) and rated the extent to which each entity possessed each type of value (*1 = not at all*, *7 = very much so*).

Moral significance. Participants responded to the same item regarding how wrong it would be to destroy the entity, as in Study 4 (0 = *not at all wrong*, to 6 = *very wrong*).

Control variables. *Mental capacity/Sentience*. Participants responded to the same questions regarding mental-capacity or sentience as in Study 1 ("Would destroying this entity negatively affect its interests?"; "To whom would it matter if this entity was destroyed—would it matter to the entity itself?"; "How much pain or suffering would this entity experience if it were destroyed") on a seven-point Likert-type scale (1 = *not at all*, to 7 = *very wrong/very much so*).

Non-intrinsic sources of value. Participants responded to the same questions about non-intrinsic sources of value, as it Studies 1 and 3 on a seven-point Likert-type scale (1 = *not at all*, to 7 = *very wrong/very much so*). This included whether destroying the entity would matter to other people ("To whom would it matter if this entity was destroyed–would it matter to other people?") and the economic value or usefulness of the entity.

Punishment. Finally, we asked participants to "Imagine that someone destroyed this entity for no reason" and to indicate deservingness of punishment ("How much would this person deserve to be punished"; 1 = *not at all*, 7 = *severely*), and punishment severity ("How painful should this person's punishment be"; 1 = *not at all*, 7 = *very painful*). Finally, to capture costly third-party punishment, participants were asked: "Imagine that you were invited to participate in an online campaign to publicly shame this person (i.e., to expose the details of this person's behaviour and make his/her identity public). How much time would you be willing to

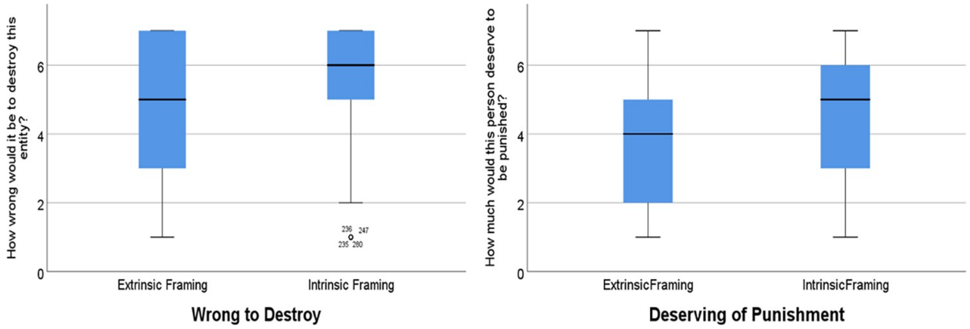

Fig 4. Destruction and punishment ratings intrinsic value vs. high extrinsic value entities (Study 5).

https://doi.org/10.1371/journal.pone.0280393.g004

invest in this campaign?" (0 = *none*, 1 = *a few minutes*, 2 = *an hour*, 3 = *a few hours*, 4 = *a day*, 5 = *two days*, 6 = *three to four days*, 7 = *five to six days*, 8 = *a week*).

Results and discussion. Our manipulations were found to be effective; participants who read about entities whose high intrinsic value was emphasized rated all entities as higher in intrinsic value ($ps < .02$) and lower in extrinsic value ($ps < .002$) than those who read about entities whose high extrinsic value was emphasized (see Table B in S1 File for means and standard deviations). We averaged across entities, thereby drawing on our entire sample, and conducted a series of *t*-tests comparing the effects of condition. This revealed that participants deemed it more wrong to destroy entities when characterized as high in intrinsic value ($M = 5.69$, $SD = 1.69$) compared to when characterized as high in extrinsic value ($M = 4.61$, $SD = 2.11$), $t(410) = 5.75$, $p < .001$, $d = 0.56$, 95% CI [.71, 1.45] (see Fig 4).

Responses also revealed participants rated deserved punishment as higher in the high-intrinsic condition ($M = 4.44$, $SD = 1.81$, vs. high-extrinsic condition; $M = 3.90$, $SD = 1.94$, $t(410) = 2.94$, $p = .004$, $d = 0.29$, 95% CI [0.80, 0.91]; see Fig 4), recommended harsher punishment in the high-intrinsic condition ($M = 3.29$, $SD = 1.76$, vs. high-extrinsic condition, $M = 2.90$, $SD = 1.19$, $t(410) = 2.22$, $p = .027$, $d = 0.26$, 95% CI [0.05, 0.73]), but did not commit more time to costly punishment in the high intrinsic condition ($M = 2.65$, $SD = 2.07$, vs. high-extrinsic condition, $M = 2.36$, $SD = 2.09$, $t(410) = 1.42$, $p = .157$, $d = 0.14$, 95% CI [0.11, -0.69]). See S1 File for analysis of control variables.

To provide further support to our *t*-test comparisons of our main dependent variable, wrongness to destroy, we conducted a 2-way ANOVA with intrinsic vs. extrinsic value conditions predicting judgements of wrongness to destroy and ratings associated with mental capacity/sentience and non-intrinsic sources of value entered as covariates in the model. The variables were averaged across entities. This revealed that the effect of value condition on judgements of wrongness to destroy remained significant, $F(8,403) = 21.36$, $p < .001$, $\eta_p^2 = .05$. Corresponding ANOVAs with deservingness of punishment or punishment severity as dependent variables revealed that value condition remained a significant predictor of deservingness of punishment, $F(8,403) = 5.04$, $p = .025$, $\eta_p^2 = .01$, but not punishment severity, $F(8,403) = 3.01$, $p = .083$, $\eta_p^2 = .01$, after controlling for ratings associated with mental capacity/sentience and non-intrinsic sources of value.

Finally, we ran regression analyses predicting wrongness to destroy and punishment for entities characterized as high in intrinsic value (i.e., those in the intrinsic properties conditions; full details are presented in S1 File). We created z-scores for all raw responses prior to averaging them across entities. This revealed when controlling for all other judgments relating to mental capacity/sentience and non-intrinsic value, ratings of intrinsic value significantly predicted wrongness to destroy ($\beta = .23$, $p < .001$), deservingness of punishment ($\beta = .22$, $p =$

.003), but not punishment severity (β = .13, p = .073) and costly third-party punishment (β = .08, p = .272).

General discussion

Across 5 studies, we provide initial evidence that people judge it morally wrong to harm things that are perceived to possess high levels of intrinsic value–entities which are beautiful, sacred, sentimental, rare, or old–and this cannot be explained merely by their usefulness or economic value. This provides novel evidence for a broader range of attributes (beyond the capacity to think, feel and hold needs and preferences alone) which may elevate the protection of environmental and cultural entities as an issue of moral significance. In our pilot studies, we find that people can reliably generate objects that they consider to be high in intrinsic value and that these are different to those objects they see as low in intrinsic value, but as valuable means to another end (such as economic value or usefulness). Furthermore, we find that people judge it more morally wrong to destroy objects high in intrinsic value (relative to objects high in extrinsic value), and that judgments of wrongness are predicted by ratings of intrinsic value. This relationship between perceived intrinsic value and wrongness of destruction can only partially be accounted for by attributions of sentience or perceived mental capacity of intrinsically valued objects or attributions of non-intrinsic value (e.g., whether it impacts negatively on another person's welfare, economic value, usefulness).

These findings uncover a broader range of properties (beyond sentience) which afford entities direct moral standing, and therefore elevates issues related to their protection as having moral significance. Supportive of this, in Study 4 we show that properties such as beauty, age, rarity, sentimentality, and sacredness predict how wrong it would be to destroy an object, and this relationship is mediated by judgments of intrinsic value. Furthermore, judgments of intrinsic value also mediated (or partially mediated) the relationship between perceived mental capacity and wrongness to destroy. This provides additional evidence that the possession of mental capacities (i.e., sentience) is but one of a range of properties that gives rise to perceived direct moral standing, and like other properties, it does so by triggering judgments of intrinsic value.

In Study 5, we expand on the findings of Study 4 to experimentally characterize the same objects as either possessing properties high in intrinsic value (e.g., beauty, age, rarity) or high in extrinsic value (e.g., usefulness). Again, we observe that people find it more morally objectionable to destroy entities when they are characterized as high in intrinsic value. Furthermore, this judgment is predicted by ratings of intrinsic value, and this explained variance cannot be accounted for by attributions of mental capacity/sentience or other sources of non-intrinsic value (as in Studies 1–3).

Our findings uncover that people base judgments of direct moral standing on properties that extend beyond the possession of mental capacities, and which therefore also extend to non-sentient objects. As such, they may inspire current theorizing in moral psychology by providng an account of how qualities beyond sentience matter for direct moral standing; people desire to protect natural and historical objects based on a range of properties that extend beyond sentience but that are nonetheless still morally relevant. Our work also provides a behavioral ethics approach to current debates in philosophy and environmental ethics, seeking to understand the basis on which lay people attribute direct moral standing to objects. In this way, it contributes to prior work seeking to understand judgments of moral action (e.g., [23, 46–48]), and properties that provide for direct moral standing (or moral considerability, see [12]). Moreover, by providing an account of how intrinsic value broadly defined contributes to direct moral standing, it also adds to work showing that people value objects differently

based on whether they are morally good or bad [49], are original [50], or that they simply exist [51].

To date, the possession of mental capacities has largely been viewed as a fundamental and necessary psychological criterion for perceived moral relevance (e.g., [10, 20]; although see [15] and [16] for broader perspectives). Other qualities such as sacredness have also been considered, but only indirectly via the introduction of concepts such as sacred value. Moreover, while such sacred value may be attached to particular entities (e.g., [21]), more often scholars have focused on 'sacred values' in describing how a set of personal values or standards are understood [24, 52, 53]. One reason for this limited focus on factors that give rise to moral standing has been the implicit assumption that morality is limited to the human domain (e.g., [29]; Universal Declaration of Human Rights). Yet as noted above, there is clearly a current trend towards expanding the limits of our moral consideration [25–29] thereby pushing us to consider other sources of direct moral standing. Our findings offer new insight into sources of perceived moral value, thereby contributing to a growing literature showing that people's moral worlds characteristically extend beyond the human domain (e.g., [18, 30, 54]). Furthermore, however, they also go beyond this past work by demonstrating that it is not only person-centric properties (e.g., mental capacity, sentience) that matter, offering instead a broad and inclusive account of why it is often considered wrong to damage or destroy, not only people and animals, but even things.

Our approach to developing this broader account of direct moral standing has been to focus on instrinsic value and to contrast this with extrinsic value. While this was an important methodoligical tactic, allowing us to examine the unqiue contribution of each type of value to judements of direct moral standing, we would note we do not see these two types of value as entirely independent. Indeed, intrinsically valuable objects such as original paintings become extrinsically valuable via their economic value. And in reverse, expensive things may be viewed as special and lead people to see them as more beautiful or sentimental (contributing to their intrinsic value). Our aim is not to completely separate these two types of value, but to show that one (intrinsic value) endows objects with moral significance, and that this effect cannot be fully account for by sources of extrinsic value (such as economic value, usefulness, or how much it might matter to another person). Our approach also makes the assumption that these two kinds of value are dimensional, rather than categorical. While this this was necessary for our statistical approach, it may be that people view these kinds of value as more categorical in their everyday judgements.

We argue that qualities giving rise to perceptions of intrinsic value confer on objects direct moral standing. This means the perceived moral wrongness of their destruction is not simply reducible to how it would impact on human needs or interests. One objection to our approach is that qualities such as beauty, and perhaps especially sentimentality, are simply in the eye of the beholder and therefore any value they add to an object is not independent of human needs or interests. We certainly acknowledge this theoretical sticking point, and it is one that has plagued theorists and researchers attempting to understand the moral and psychological basis for conservation (see [34–38]; also see below). Nonetheless, from a psychological perspective, this same issue bleeds into work on the role of mind perception in providing for direct moral standing. There is now a significant body of work in psychology showing that perceiving the minds of others increases our care and concern for those others, and that denying this capacity reduces that concern (see [20]). Critically, however, this research also tells us that it is often our own needs and interests that dictate when, and to what extent, we are likely to percieve the mental attributes of others (see [18, 55]), suggesting that moral standing based on more traditional foundations of sentience or mental capacity is also not entirely seperable, in a psychological sense, from the needs and interests of the perceiver.

Our findings have implications for our understanding of the psychology of conservation and protection. Those who destroy the natural environment are viewed as morally questionable [56–59], demonstrating that such actions are understood as having moral consequences. Yet, where prior work has attempted to measure environmental concern in ways that are independent of a consideration of human needs [60, 61], it has been met with limited success. Stern and Dietz [58] measured both altruistic values (focused on other human needs met by conservation efforts–means-end value perspective), as well as biospheric values (focused on the need to protect the environment for its own sake–intrinsic value perspective), but found that these two value orientations could not be meaningfully differentiated. More recently, Bastian, Brewer, Duffy, & Van Lange [62] found that making harm salient to even minimally morally relevant creatures (crickets) was enough to increase cooperation within a resource dilemma, yet this study limited its focus to (minimally) sentient creatures. Most closely aligned to our endevour here are recent findings showing that perceptions of beauty contribute to the direct moral standing of people, animals, the environment, and even buildings [63, 64]. While this is the first work to show that aesthetic qualities may contribute to direct moral standing, it focuses on just one contributor to intrinsic value. Our findings help to move beyond these limitations, clearly demonstrating that people do attribute direct moral standing to cultural and environmental objects; that they do this based on the possession of a number of candidate characteristics (e.g., beauty, rarity, sacredness, sentimentality, or age) and which extend beyond a focus on sentience; and that these characteristics give rise to intrinsic value, and it is this type of value which explains judgements of direct moral standing, including when controlling for sources of non-intrinsic value (e.g., the needs of humans).

In this way, our work also offers important insights into practical ways to elevate the moral significance of issues of conservation and protection. Although altruistic values (our concern for future humans) provide an important avenue through which to trigger the motivation to protect the natural and cultural environment, our findings highlight additional avenues which together with altruistic approaches may increase the utility of current interventions. For instance, our findings offer insight into the specific properties which may be emphasized in attempts to raise issues of protection to moral significance. Focusing people on qualities such as the beauty, age, or rarity of environmental and cultural objects may not only increase altruistic motivations for protection (so future humans can enjoy these entities), but also biospheric motivations–that is a desire to protect these things independent of their means-end value for other humans.

Limitations

The current studies have aimed to explore a new direction in our understanding of the psychology behind moral standing, and reasons for protecting cultural and environmental objects. In this way, our aim was to lay the groundwork for future research. Nonetheless, there are some limitations to our approach. First, we drew on online samples and focused on a number of relatively abstract questions and concepts. We see this as a necessary first step but would expect to see more concrete applications of these ideas in future studies. Second, we developed a set of exemplars based on pilot studies in order to limit the idiosyncratic effects of specific entities. Certainly, some qualities will matter more for some entities than others; for instance, it is likely a sea turtle's capacity for sentience that matters most, but a paintings beauty or rarity. This same approach was used in Study 5 where we manipulated perceptions of objects based on a range of intrinsic properties identified as giving rise to intrinsic value (in Study 4). While our findings cannot determine which qualities matter the most for which entities, our findings offer an important foundation for future research; they identify that an entity's

intrinsic value, and in turn its direct moral standing, can be influenced by a range of properties that go beyond mental capacity or sentience. Third, we asked people how wrong it would be to destroy each of the entities, rather than how morally wrong it would be to destroy them. While we feel confident that our measurement approach was capturing moral considerations (which commonly centre on harm), future research might seek to employ a more direct measure of moral standing.

Concluding remarks

Issues of protection and conservation are of critical importance. This may include specific forms of cultural heritage that have no real price tag, but are precious because of rarity, beauty or irreplaceability. More pressing, it may include issues related to climate change, raising the possibility that people may be motivated to take climate action because they value the protection of nature for its own sake–for example, if not for reasons following from climate change, some people may be persuaded to help protect a rainforest for intrinsic reasons such as its rarity, beauty, or irreplaceability. More generally, understanding avenues through which to elevate the moral significance of these issues offers important insights into how to conduct behavior change campaigns.

We see our findings as providing initial insight into a broader and more inclusive source of direct moral standing, and therefore a basis from which we can begin to build a psychology around environmental ethics; that is, a perspective which recognizes that we are often concerned about non-human entities and resources because we believe that they are worthy of protection, not simply for our own sake, but also for theirs.

Supporting information

S1 File. Supplementary information.
(DOCX)

Acknowledgments

We would like to thank Geoffrey Goodwin for his significant feedback and review on various versions of this manuscript.

Author Contributions

Conceptualization: Brock Bastian, Charlie R. Crimston, Paul A. M. van Lange.

Data curation: Brock Bastian, Charlie R. Crimston, Christoph Klebl.

Formal analysis: Brock Bastian, Christoph Klebl.

Funding acquisition: Brock Bastian, Paul A. M. van Lange.

Investigation: Charlie R. Crimston, Paul A. M. van Lange.

Project administration: Brock Bastian, Charlie R. Crimston, Paul A. M. van Lange.

Resources: Charlie R. Crimston.

Software: Charlie R. Crimston.

Supervision: Brock Bastian, Paul A. M. van Lange.

Validation: Christoph Klebl.

Writing – original draft: Brock Bastian.

Writing – review & editing: Charlie R. Crimston, Christoph Klebl, Paul A. M. van Lange.

References

1. Johnson I. Why extreme Islamists are intent on destroying cultural artifacts? 2013 [cited 2020 April 6]. Available from: https://urbanintellectuals.com/why-extreme-islamists-are-intent-on-destroying-cultural-artifacts/.
2. Rashid A. After 1,700 years, Buddhas fall to Taliban dynamite 2001 [cited 2016 September 23]. Available from: https://www.telegraph.co.uk/news/worldnews/asia/afghanistan/1326063/After-1700-years-Buddhas-fall-to-Taliban-dynamite.html.
3. Simons M. Prison sentence over smashing of shrins in Timbuktu: 9 years 2016 [cited 2016 October 5]. Available from: https://www.nytimes.com/2016/09/28/world/europe/ahmad-al-faqi-al-mahdi-timbuktu-mali.html?_r=0.
4. Team NN. Ecuador grants rights to nature 2008 [cited 2020 April 8]. Available from: http://blogs.nature.com/news/2008/09/ecuador_grants_rights_to_natur.html.
5. McCarthy J., River national park in New Zealand are now legally people 2016 [cited 2016 July 16]. Available from: https://www.globalcitizen.org/en/content/new-zealand-nature-personhood-climate-change/.
6. Bentham J. The principles of morals and legislation. London: Printed for T Payne (Republished 1988 New York Prometheus Books). 1789.
7. Mill JS. Utilitarianism. Crisp R, editor. Oxford: Oxford University Press; 1998.
8. Sidgwick H. The methods of ethics. Indianapolis: Hackett Publishing; 1907.
9. Kant I. Groundwork of the metaphysics of morals. New York: Liberal Arts Press; 1785/1959.
10. Gray HM, Gray K, Wegner DM. Dimensions of mind perception. Science. 2007; 315(5812):619. Epub 2007/02/03. https://doi.org/10.1126/science.1134475 PMID: 17272713.
11. Singer P. Animal liberation. Australia: Random House; 1995.
12. Goodwin GP. Experimental approaches to moral standing. Philosophy Compass. 2015; 10(12):914–26. https://doi.org/10.1111/phc3.12266
13. Jack AI, Robbins P. The phenomenal stance revisited. Review of Philosophy and Psychology. 2012; 3 (3):383–403. https://doi.org/10.1007/s13164-012-0104-5
14. Knobe J, Prinz J. Intuitions about consciousness: Experimental studies. Phenomenology and the Cognitive Sciences. 2008; 7(1):67–83. https://doi.org/10.1007/s11097-007-9066-y
15. Piazza J, Landy JF, Goodwin GP. Cruel nature: Harmfulness as an important, overlooked dimension in judgments of moral standing. Cognition. 2014; 131(1):108–24. https://doi.org/10.1016/j.cognition.2013.12.013 PMID: 24462925
16. Sytsma J, Machery E. On the relevance of folk intuitions: A commentary on Talbot. Consciousness and Cognition. 2012; 21(2):654–60. https://doi.org/10.1016/j.concog.2011.04.003 PMID: 21570319
17. Bastian B, Laham SM, Wilson S, Haslam N, Koval P. Blaming, praising, and protecting our humanity: the implications of everyday dehumanization for judgments of moral status. Br J Soc Psychol. 2011; 50 (3):469–83. Epub 2011/09/03. https://doi.org/10.1348/014466610x521383 PMID: 21884545.
18. Bastian B, Loughnan S. Resolving the meat-paradox: A motivational account of morally troublesome behavior and its maintenance. Pers Soc Psychol Rev. 2017; 21(3):278–99. Epub 2016/05/22. https://doi.org/10.1177/1088868316647562 PMID: 27207840.
19. Feinberg J. The rights of animals and unborn generations. In: Blackstone W, editor. Philosophy and Environmental Crisis. Georgia: University of Georgia Press; 1974.
20. Gray K, Young L, Waytz A. Mind perception is the essence of morality. Psychological Inquiry. 2012; 23 (2):101–24. https://doi.org/10.1080/1047840X.2012.651387 PMID: 22754268
21. Frimer JA, Tell CE, Haidt J. Liberals condemn sacrilege too: The harmless desecration of Cerro Torre. Social Psychological and Personality Science. 2015; 6(8):878–86. https://doi.org/10.1177/1948550615597974
22. Ginges J, Atran S, Medin D, Shikaki K. Sacred bounds on rational resolution of violent political conflict. Proceedings of the National Academy of Sciences. 2007; 104(18):7357–60. https://doi.org/10.1073/pnas.0701768104 PMID: 17460042
23. Graham J, Haidt J, Nosek BA. Liberals and conservatives rely on different sets of moral foundations. J Pers Soc Psychol. 2009; 96(5):1029–46. Epub 2009/04/22. https://doi.org/10.1037/a0015141 PMID: 19379034.
24. Tetlock PE. Thinking the unthinkable: Sacred values and taboo cognitions. Trends in Cognitive Sciences. 2003; 7(7):320–4. https://doi.org/10.1016/s1364-6613(03)00135-9 PMID: 12860191

25. Lecky WEH. History of European morals, from Augustus to Charlemagne. London, UK: Longmans Green And Co; 1869.
26. Pinker S, editor. The better angels of our nature: Why violence has declined. New York: Viking; 2011.
27. Singer P. The expanding circle: Ethics and sociobiology. New York: Farrar, Straus & Giroux; 1981.
28. Glover J. Humanity: A moral history of the twentieth century. New Haven: Yale University Press; 1999.
29. Sapontzis SF. A critique of personhood. Ethics. 1981; 91(4):607–18. https://doi.org/10.1086/292273
30. Crimston D, Bain PG, Hornsey MJ, Bastian B. Moral expansiveness: Examining variability in the extension of the moral world. J Pers Soc Psychol. 2016; 111(4):636–53. Epub 2016/01/12. https://doi.org/10.1037/pspp0000086 PMID: 26751743.
31. Callicott JB. Animal liberation: A triangular affair. Environmental ethics. 1980; 2(4):311–38. https://doi.org/10.5840/enviroethics19802424
32. Goodpaster KE. On being morally considerable. The Journal of Philosophy. 1978; 75(6):308–25. https://doi.org/10.2307/2025709
33. Leopold A. A sand county almanac, and sketches here and there. Oxford: Oxford University Press; 1949.
34. Naess A. The shallow and the deep, long-range ecology movement. A summary. Inquiry. 1973; 16(1–4):95–100. https://doi.org/10.1080/00201747308601682
35. Routley R. Is there a need for a new, an environmental ethic? Proceedings of the XVth World Congress of Philosophy. 1973; 1:205–10. https://doi.org/10.5840/wcp151973136
36. Stone CD. Should trees have standing? Southern California Law Review. 1972; 45:450–501.
37. Brennan A, Lo Y. Environmental ethics. The Stanford Encyclopedia of Philosophy [Internet]. 2015. Available from: https://plato.stanford.edu/entries/ethics-environmental/.
38. Vucetich JA, Bruskotter JT, Nelson MP. Evaluating whether nature's intrinsic value is an axiom of or anathema to conservation. Conservation Biology. 2015; 29(2):321–32. https://doi.org/10.1111/cobi.12464 PMID: 25704250
39. Bradley B. Two concepts of intrinsic value. Ethical Theory and Moral Practice. 2006; 9(2):111–30. https://doi.org/10.1007/s10677-006-9009-7
40. Rabinowicz W, Rønnow-Rasmussen T. II—A distinction in value: Intrinsic and for its own sake. Proceedings of the Aristotelian Society. 2000; 100(1):33–51. https://doi.org/10.1111/j.0066-7372.2003.00002.x
41. Faul F, Erdfelder E, Buchner A, Lang A-G. Statistical power analyses using G*Power 3.1: Tests for correlation and regression analyses. Behavior Research Methods. 2009; 41(4):1149–60. https://doi.org/10.3758/BRM.41.4.1149 PMID: 19897823
42. Zimmerman MJ. Intrinsic vs. extrinsic value. The Stanford Encyclopedia of Philosophy [Internet]. 2015. Available from: https://plato.stanford.edu/archives/spr2015/entries/value-intrinsic-extrinsic/.
43. Tetlock PE, Kristel OV, Elson SB, Green MC, Lerner JS. The psychology of the unthinkable: Taboo trade-offs, forbidden base rates, and heretical counterfactuals. Journal of Personality and Social Psychology. 2000; 78(5):853–70. https://doi.org/10.1037//0022-3514.78.5.853 PMID: 10821194
44. Hayes AF. Beyond Baron and Kenny: Statistical mediation analysis in the new millennium. Communication Monographs. 2009; 76(4):408–20. https://doi.org/10.1080/03637750903310360
45. Hayes AF. Introduction to mediation, moderation, and conditional process analysis: A regression-based approach: Guilford Publications; 2017.
46. Greene JD, Sommerville RB, Nystrom LE, Darley JM, Cohen JD. An fMRI investigation of emotional engagement in moral judgment. Science. 2001; 293(5537):2105–8. https://doi.org/10.1126/science.1062872 PMID: 11557895
47. Haidt J. The emotional dog and its rational tail: A social intuitionist approach to moral judgment. Psychological Review. 2001; 108(4):814–34. https://doi.org/10.1037/0033-295x.108.4.814 PMID: 11699120
48. Schein C, Gray K. The unifying moral dyad: Liberals and conservatives share the same harm-based moral template. Personality and Social Psychology Bulletin. 2015; 41(8):1147–63. https://doi.org/10.1177/0146167215591501 PMID: 26091912
49. Jarudi I, Kreps T, Bloom P. Is a refrigerator good or evil? The moral evaluation of everyday objects. Social Justice Research. 2008; 21(4):457–69. https://doi.org/10.1007/s11211-008-0082-z
50. Newman GE, Bloom P. Art and authenticity: The importance of originals in judgments of value. Journal of Experimental Psychology: General. 2012; 141(3):558–69. https://doi.org/10.1037/a0026035 PMID: 22082113
51. Eidelman S, Crandall CS, Pattershall J. The existence bias. Journal of Personality and Social Psychology. 2009; 97(5):765–75. https://doi.org/10.1037/a0017058 PMID: 19857000

52. Atran S, Ginges J. Religious and sacred imperatives in human conflict. Science. 2012; 336(6083):855–7. https://doi.org/10.1126/science.1216902 PMID: 22605762

53. Haidt J, Graham J. When morality opposes justice: Conservatives have moral intuitions that liberals may not recognize. Social Justice Research. 2007; 20(1):98–116. https://doi.org/10.1007/s11211-007-0034-z

54. Amiot CE, Bastian B. Toward a psychology of human–animal relations. Psychological Bulletin. 2015; 141(1):6–47. https://doi.org/10.1037/a0038147 PMID: 25365760

55. Castano E, Giner-Sorolla R. Not quite human: Infrahumanization in response to collective responsibility for intergroup killing. Journal of Personality and Social Psychology. 2006; 90(5):804–18. https://doi.org/10.1037/0022-3514.90.5.804 PMID: 16737374

56. Feinberg M, Willer R. The moral roots of environmental attitudes. Psychol Sci. 2013; 24(1):56–62. Epub 2012/12/12. https://doi.org/10.1177/0956797612449177 PMID: 23228937.

57. Markowitz E. Is climate change an ethical issue? Examining young adults' beliefs about climate and morality. Climatic Change. 2012;114. https://doi.org/10.1007/s10584-012-0422-8

58. Stern PC, Dietz T. The value basis of environmental concern. Journal of Social Issues. 1994; 50(3):65–84. https://doi.org/10.1111/j.1540-4560.1994.tb02420.x

59. Van Lange PAM, Joireman J, Milinski M. Climate change: What psychology can offer in terms of insights and solutions. Current Directions in Psychological Science. 2018; 27(4):269–74. https://doi.org/10.1177/0963721417753945 PMID: 30166778

60. Dunlap RE, Van Liere KD. The "New Environmental Paradigm". The Journal of Environmental Education. 1978; 9(4):10–9. https://doi.org/10.1080/00958964.1978.10801875

61. Stern PC, Dietz T, Guagnano GA. The new ecological paradigm in social-psychological context. Environment and Behavior. 1995; 27(6):723–43. https://doi.org/10.1177/0013916595276001

62. Bastian B, Brewer M, Duffy J, Van Lange PAM. From cash to crickets: The non-monetary value of a resource can promote human cooperation. Journal of Environmental Psychology. 2019; 61:10–9. https://doi.org/10.1016/j.jenvp.2018.11.002

63. Klebl C, Luo Y, Bastian B. Beyond aesthetic judgment: Beauty increases moral standing through perceptions of purity. Personality and Social Psychology Bulletin. 2021:01461672211023648. https://doi.org/10.1177/01461672211023648 PMID: 34167390

64. Klebl C, Luo Y, Tan NP-J, Ping Ern JT, Bastian B. Beauty of the Beast: Beauty as an important dimension in the moral standing of animals. Journal of Environmental Psychology. 2021; 75:101624. https://doi.org/10.1016/j.jenvp.2021.101624
